# Prognostic significance of continuing immunotherapy beyond progression in unresectable lung adenocarcinoma

**DOI:** 10.3389/fimmu.2026.1805604

**Published:** 2026-07-08

**Authors:** Fang Hu, Liang Zheng, Haoming Xu, Feng Pan, Wei Nie, Runbo Zhong, Yuqing Lou, Baohui Han, Xiaoxuan Zheng, Hua Zhong, Xueyan Zhang

**Affiliations:** 1 Department of Respiratory and Critical Care Medicine, Shanghai Chest Hospital, Shanghai Jiao Tong University School of Medicine, Shanghai, China; 2 Department of Thoracic Medical Oncology, Zhejiang Cancer Hospital, Hangzhou Institute of Medicine (HIM), Chinese Academy of Sciences, Hangzhou, Zhejiang, China; 3 Department of Respiratory Endoscopy, Shanghai Chest Hospital, Shanghai Jiao Tong University School of Medicine, Shanghai, China

**Keywords:** cross-line immunotherapy, immune checkpoint inhibitors, lung adenocarcinoma, nomogram, prognosis

## Abstract

**Background:**

In patients with unresectable lung adenocarcinoma, whether cross-line immunotherapy (CIT) can improve clinical outcomes remains unclear.

**Methods:**

This study enrolled patients with unresectable lung adenocarcinoma who received immune checkpoint inhibitors (ICIs)–based therapy and subsequently experienced disease progression. According to post-progression treatment strategies, patients were categorized into two groups: CIT group and Non-CIT group. The primary endpoints were second progression-free survival (PFS2), overall survival (OS), and post-progression survival (PPS), analyzed both using unadjusted and inverse probability for treatment weighting (IPTW) analyses. Survival outcomes were analyzed using the Kaplan–Meier method and compared with the log-rank test. Multivariate Cox regression was performed to identify independent prognostic factors. A nomogram for PPS prediction was built and internally validated with bootstrap resampling and receiver operating characteristic (ROC) curves.

**Results:**

A total of 185 patients met the inclusion criteria and were studied assessed, including 77 in the CIT group and 108 in the Non-CIT group. Baseline characteristics were generally balanced between two groups. After IPTW adjustment, median PFS2 was significantly longer in the CIT group compared with the Non-CIT group (6.3 vs. 2.8 months; hazard ratio (HR)=0.54, 95% confidence intervals (CI): 0.39-0.77, P<0.001). Similarly, the CIT group demonstrated improved median PPS and OS compared with Non-CIT group. Multivariate Cox analysis also identified CIT as independent predictors of PFS2, PPS and OS. The PPS nomogram showed good predictive performance.

**Conclusions:**

In patients with unresectable lung adenocarcinoma who progressed after initial immunotherapy, continuation of immunotherapy beyond progression was associated with significantly improved survival. The PPS nomogram may facilitate risk stratification and personalized post-progression management.

## 1 Introduction

Lung adenocarcinoma, the most prevalent histological subtype of non-small cell lung cancer (NSCLC), has witnessed a paradigm shift in its therapeutic landscape with the advent of immune checkpoint inhibitors (ICIs) targeting the programmed death receptor 1/programmed death receptor ligand 1 (PD-1/PD-L1) axis (1). The success of first-line ICI-based combinations, either with chemotherapy or anti-angiogenic agents, has established immunotherapy as a cornerstone for advanced driver-gene wild-type disease, significantly improving survival outcomes for a substantial proportion of patients (2, 3). However, treatment selection for unresectable or metastatic lung adenocarcinoma is no longer determined solely by treatment line, but is increasingly guided by comprehensive molecular profiling, PD-L1 expression, prior treatment exposure, and patient-level clinical factors. For patients harboring actionable alterations such as EGFR, ALK, ROS1, BRAF, MET exon 14 skipping, RET, NTRK, KRAS G12C, or ERBB2/HER2, treatment generally follows biomarker-matched targeted strategies; whereas for patients without actionable driver alterations, immunotherapy-based regimens, with or without chemotherapy or anti-angiogenic therapy, constitute a major therapeutic backbone (4–6).

Although immunotherapy has substantially improved outcomes for patients with unresectable lung adenocarcinoma lacking actionable driver alterations, most patients treated with ICI monotherapy or ICI-based combination regimens eventually experience disease progression. Treatment decision-making after progression should not be viewed simply as a fixed sequential switch from one line of therapy to the next (7, 8). Instead, it requires an individualized assessment of the tempo and extent of progression, the duration of prior clinical benefit from immunotherapy, treatment tolerability, performance status, residual disease burden, and the feasibility of reassessing tumor biology through tissue- or plasma-based testing (9). In selected patients with oligo-progressive disease, local ablative therapy combined with continuation of an otherwise effective systemic regimen may also represent an adaptive treatment strategy (10). Therefore, although treatment lines remain useful for defining clinical-trial populations and summarizing prior treatment exposure, they do not fully capture the dynamic decision-making that characterizes contemporary management of lung adenocarcinoma. Unlike cytotoxic agents, ICIs do not primarily exert their antitumor effects through direct tumor-cell killing, but rather through the release of inhibitory immune checkpoints and the reinvigoration of pre-existing T-cell-mediated antitumor immunity. The magnitude and durability of this response may be shaped by multiple components of the cancer-immunity cycle, including tumor antigen presentation, T-cell priming and infiltration, the immunosuppressive tumor microenvironment, and the immunomodulatory effects of subsequent combination partners. Therefore, in selected patients, radiographic disease progression may not invariably indicate an immediate or complete loss of ongoing immune modulation (11, 12). This biological rationale underpins the emerging concept of “cross-line immunotherapy”, wherein the ICI component is continued beyond progression while combining it with a new therapeutic partner. Continuing PD-1/PD-L1 inhibition may sustain a foundational anti-tumor immune tone, while the addition of a new agent (e.g., a different chemotherapy regimen, anti-angiogenic drug, or novel investigational agent) could remodel the tumor microenvironment, overcome emergent resistance mechanisms, and synergistically re-invigorate the immune response (13–15).

From an immuno-oncology perspective, the effect of ICIs reflects a dynamic interaction between tumor cells, antigen presentation, effector T-cell infiltration, immune checkpoints, and the broader tumor microenvironment. Resistance to PD-1/PD-L1 blockade may arise through multiple mechanisms, including impaired antigen presentation, loss or dysfunction of effector T-cell activity, activation of alternative immune-suppressive pathways, immune-excluded or immune-desert phenotypes, and oncogenic or co-mutational programs such as STK11/KEAP1 alterations in lung adenocarcinoma (16, 17). Therefore, the benefit of immunotherapy beyond progression is likely to be heterogeneous and dependent on patient and tumor selection. PD-L1 expression remains the most widely used clinical biomarker for selecting patients for ICI-based therapy in NSCLC, while tumor mutational burden, tumor-infiltrating lymphocytes, antigen-presentation machinery, circulating tumor DNA dynamics, and specific genomic co-alterations are being investigated as complementary or emerging biomarkers (18). However, no single biomarker has been sufficiently validated to guide continuation of immunotherapy beyond progression, highlighting the need for clinically applicable predictive models.

While a few small-scale studies in NSCLC and real-world evidence in other cancer cohorts have hinted at the potential survival benefit of cross-line immunotherapy (19–21). However, available clinical evidence remains inconsistent. An exploratory analysis of the OAK trial suggested that selected patients receiving atezolizumab beyond progression might achieve prolonged survival (22). However, a randomized phase II trial showed that continuing pembrolizumab with additional chemotherapy after progression on prior PD-1/PD-L1 inhibitor monotherapy did not provide a broad clinical benefit (23). In contrast, Lung-MAP S1800A demonstrated improved overall survival with ramucirumab plus pembrolizumab compared with standard-of-care therapy in patients with NSCLC previously treated with immunotherapy (24). These heterogeneous findings suggest that immunotherapy beyond progression may not be appropriate for all patients and highlight the importance of identifying patients most likely to benefit (24). Understanding which patients may benefit from immunotherapy beyond initial progression remains important for refining individualized treatment strategies and improving outcomes, although prospective biomarker-stratified evidence to guide such selection remains limited.

Against this backdrop, the prognostic factors for the efficacy of continued immunotherapy following progression remain unclear, and there are currently no validated models available to guide patient selection. Investigating the association between patient characteristics, tumor features, and immunotherapy response could enable the development of a predictive framework to identify patients most likely to derive benefit. Such an approach would not only address a significant clinical knowledge gap but also inform evidence-based strategies for managing unresectable lung adenocarcinoma.

Therefore, we conducted this retrospective study to evaluate the impact of cross-line immunotherapy on survival outcomes in patients with unresectable lung adenocarcinoma and to explore clinical predictors of benefit. Our findings are intended to provide real-world evidence for this common clinical scenario and to inform future efforts to refine patient-selection strategies in unresectable lung adenocarcinoma.

## 2 Materials and methods

### 2.1 Patients

The medical records of patients with unresectable stage III–IV or recurrent lung adenocarcinoma who received ICIs (including anti-PD-1 and anti-PD-L1 therapy) at Shanghai Chest Hospital between October 2017 and March 2022 were screened and reviewed. This study was approved by the Institutional Review Board of Shanghai Chest Hospital (No. LS1808, Shanghai, China).

Inclusion criteria included: (1) adult patients (≥18 years old) histologically or cytologically confirmed primary lung adenocarcinoma with unresectable locally advanced, recurrent, or metastatic disease; (2) patients who experienced disease progression after treatment with ICIs; (3) with measurable target lesions; (4) clinical TNM staging was assigned according to the 8th edition of the AJCC classification system based on all available clinical, radiologic, and pathological information. Baseline staging evaluations included chest computed tomography, abdominal ultrasound or computed tomography, brain magnetic resonance imaging, and bone scans; positron emission tomography–computed tomography was performed when clinically indicated or available. Pathological confirmation of nodal disease was obtained in selected patients when accessible or clinically indicated, including supraclavicular lymph node biopsy and endobronchial ultrasound-guided transbronchial needle aspiration for mediastinal lymph nodes. However, systematic invasive mediastinal staging using EBUS or EUS was not performed in all patients; therefore, nodal status was classified as clinical nodal stage incorporating pathological confirmation where available; (5) ECOG performance status ≤ 2. Firstly, patients who had received immunotherapy were screened. Then, patients with lung adenocarcinoma with sufficient clinical data were selected. Initially, patients with lung adenocarcinoma who had received immunotherapy and had complete clinical data were identified. Subsequently, those who experienced disease progression following immunotherapy were selected. Finally, patients were categorized based on post-progression treatment patterns for comparative analysis.

Exclusion criteria included: (1) histologic subtypes other than adenocarcinoma; (2) cases with unclassified or indeterminate pathological types; (3) tumors originating from organs other than the lung, to avoid inclusion of metastatic non-pulmonary adenocarcinoma; (4) harbored sensitizing EGFR mutations, ALK rearrangements, or ROS1 fusions; (5) insufficient survival data; (6) absence of regular systemic therapy; (7) coexistence of another malignancy; (8) serious systemic comorbidities, including active severe autoimmune diseases that could interfere with treatment or follow-up; and (9) loss to follow-up.

### 2.2 Data collection

The follow-up was carried out via hospital visits or phone calls. Major clinicopathological factors, such as age, gender, smoking history, TNM stage, gene mutation status, primary lung lesion status, tumor location, brain metastasis, liver metastasis, PD-L1 expression, line of immunotherapy, immunotherapy regimen, immune-related adverse events (irAEs) were collected. Prior primary lung lesion resection referred to surgical resection performed before the development of unresectable recurrent or metastatic disease. All patients included in this study had unresectable disease at the time of ICI treatment and subsequent post-progression treatment evaluation. Unavailable data were marked as missing (NA, not available). For variables with clinically meaningful missingness, such as PD-L1 expression, an “unknown” category was retained and included in the analyses to avoid excluding patients from survival models. No multiple imputation was performed because of the retrospective design and limited sample size. PD-L1 expression was assessed by immunohistochemistry in tumor tissue using clinically available assays with 22C3, E1L3N, or SP142 antibodies, according to routine clinical practice during the study period. A tumor proportion score or tumor-cell staining threshold of 1% or higher was defined as PD-L1 positive. PD-L1 expression was assessed using different clinically available assays, which may have introduced inter-assay variability.

Molecular testing was performed as part of routine clinical practice using available tumor tissue, cytology specimens, or plasma samples when tissue was insufficient. Genetic alterations were detected using amplification refractory mutation system polymerase chain reaction (ARMS-PCR) or targeted next-generation sequencing (NGS), according to clinical availability during the study period. Briefly, ARMS-PCR is an allele-specific PCR-based method in which amplification occurs preferentially when primers match predefined mutant alleles. This approach has high analytical sensitivity for known hotspot alterations included in the assay, but its scope is limited to prespecified variants and it does not provide broad genomic profiling (25). In contrast, targeted NGS allows simultaneous interrogation of multiple genes and broader classes of genomic alterations, including single-nucleotide variants, insertions/deletions, copy-number alterations, and gene fusions, depending on the panel design (26). Therefore, patients tested only by ARMS-PCR may have had less comprehensive molecular characterization than those tested by NGS.

### 2.3 Treatment and follow-up

All patients with unresectable lung adenocarcinoma included in this study received at least one cycle of ICIs–based therapy. The included immunotherapeutic agents differed in target, dosing schedule, administration method, approval status, and clinical availability. PD-1 inhibitors included nivolumab, pembrolizumab, tislelizumab, sintilimab, and camrelizumab, whereas PD-L1 inhibitors included atezolizumab and durvalumab. Detailed information on each immunotherapeutic agent, including drug name, target, dose and usage, and administration method, is provided in **Supplementary Additional Table 1**. The selection of specific ICI agents and combination regimens was determined by treating physicians according to drug availability, regulatory approval, reimbursement status, patient preference, toxicity profile, and clinical practice during the study period. Because the objective of this study was to evaluate the overall treatment strategy of continuing PD-1/PD-L1 blockade beyond progression rather than the comparative efficacy of individual ICI agents or specific combinations, patients receiving different anti-PD-1 or anti-PD-L1 agents and ICI-based regimens were included. Treatment-related variables, including immunotherapy regimen and line of immunotherapy, were included in the adjustment analyses to reduce potential bias related to treatment heterogeneity. Treatment was continued until radiographic or clinical disease progression, unacceptable toxicity, or death. After progression on prior ICIs-based therapy, patients were divided into two groups: those who continued immunotherapy in subsequent treatment lines (cross-line immunotherapy, CIT group) and those who did not (Non-CIT group). CIT was defined as receipt of any ICI-containing systemic regimen after first documented progression within the predefined 8-week decision window, whereas Non-CIT was defined as subsequent systemic therapy without ICIs. The ICI used after progression could be the same as or different from the pre-progression ICI, according to the treating physician’s discretion. Radiotherapy was not included as a baseline covariate because it could occur at different stages of the disease course and varied substantially in timing, site, intent, dose-fractionation, and frequency. Given the incomplete and heterogeneous radiotherapy records in this retrospective cohort, a simple binary classification was considered insufficient to reliably assess its impact on survival outcomes. Follow-up assessments included physical examination, imaging, and routine laboratory tests every 6–8 weeks. To reduce potential immortal time bias, subsequent treatment strategy was determined within a predefined 8-week decision window after first documented disease progression, corresponding to the routine 6–8-week assessment interval in our clinical practice. Patients who subsequently received ICI-containing regimens within this window were assigned to the CIT group, whereas those who received systemic therapy without ICIs were assigned to the Non-CIT group. Treatment decisions occurring beyond this window were not used to redefine group assignment. Treatment response was evaluated according to the Response Evaluation Criteria in Solid Tumors (RECIST) version 1.1.

Because all patients included in this study had documented disease progression after prior ICI-based therapy, Progression-free survival 1 (PFS1) was defined as the time from initiation of initial ICI-based therapy to first documented disease progression. Progression-free survival 2 (PFS2) was defined as the time from initiation of subsequent post-progression treatment to second documented disease progression or death from any cause, whichever occurred first; patients alive without second progression were censored at last follow-up. Post-progression survival (PPS) was defined as the time from first documented disease progression to death from any cause, with patients alive at last follow-up censored. Overall survival (OS) was defined as the time from initiation of initial ICI-based therapy to death from any cause, with patients alive at last follow-up censored. The primary endpoints were PFS2, PPS, and OS. The median follow-up time, calculated using the reverse Kaplan–Meier method, was 54.1 months.

### 2.4 Nomogram development and validation

The nomogram was constructed based on the regression coefficients from the final multivariate Cox model. Each variable was assigned points proportional to its regression coefficient, with the total points corresponding to predicted survival probabilities at 6, 12, and 24 months. The discriminative ability of the nomogram was evaluated using Harrell’s concordance index (C-index), which measures the probability that the model correctly ranks pairs of patients according to their survival times. Internal validation was performed using bootstrap resampling with 500 iterations to assess model stability and reduce overfitting bias. The bootstrap-corrected C-index was calculated to provide an unbiased estimate of model performance. Model calibration was evaluated by comparing predicted survival probabilities with observed survival rates using calibration plots at 6, 12 and 24 months. Patients were stratified into three risk groups (low, medium, and high) based on tertiles of the nomogram-derived risk scores. Kaplan-Meier survival curves were constructed for each risk group, and differences were assessed using the log-rank test. Multicollinearity among the variables included in the nomogram was assessed using variance inflation factors (VIF). VIF values > 2.0 were interpreted as indicating the presence of multicollinearity. All 7 variables in the final nomogram showed VIF values <2.0, indicating no significant multicollinearity issues. The highest VIF was observed for PD-L1 expression status (VIF = 1.26), both well below the threshold for concern. Bootstrap resampling was used solely for internal validation of the prognostic nomogram and was not intended to address confounding related to non-random treatment allocation.

### 2.5 Statistical analysis

All statistical analyses were performed using Python 3.9.6 with the lifelines package (version 0.27.7) for survival analysis. Continuous variables were expressed as medians with interquartile ranges, while categorical variables were summarized as frequencies and percentages. Differences between categorical variables were compared using the Chi-square or Fisher’s exact test, as appropriate. To address potential treatment-selection bias arising from the non-random allocation of post-progression therapy, propensity score-based inverse probability of treatment weighting (IPTW) was performed as an additional adjustment analysis. The propensity score was estimated using a multivariable logistic regression model including clinically relevant baseline variables that could influence the decision to continue immunotherapy beyond progression. Stabilized weights were applied to create a weighted pseudo-population in which measured baseline characteristics were balanced between the CIT and Non-CIT groups. Covariate balance before and after IPTW was assessed using standardized mean differences (SMDs), with an absolute SMD <0.10 considered indicative of acceptable balance. IPTW-weighted Kaplan–Meier curves and weighted Cox proportional hazards models with robust standard errors were used to evaluate survival outcomes after adjustment for measured confounding. Both unweighted and IPTW-weighted analyses were performed, with the IPTW-weighted results considered the primary adjusted analyses. Kaplan–Meier curves and log-rank tests were used for univariate survival comparisons, and Cox proportional hazards models were used to estimate hazard ratio (HR) and 95% confidence interval (CI). All statistical tests were two-sided, and a p-value <0.05 was considered statistically significant.

## 3 Results

### 3.1 Patient characteristics

**Figure 1:**
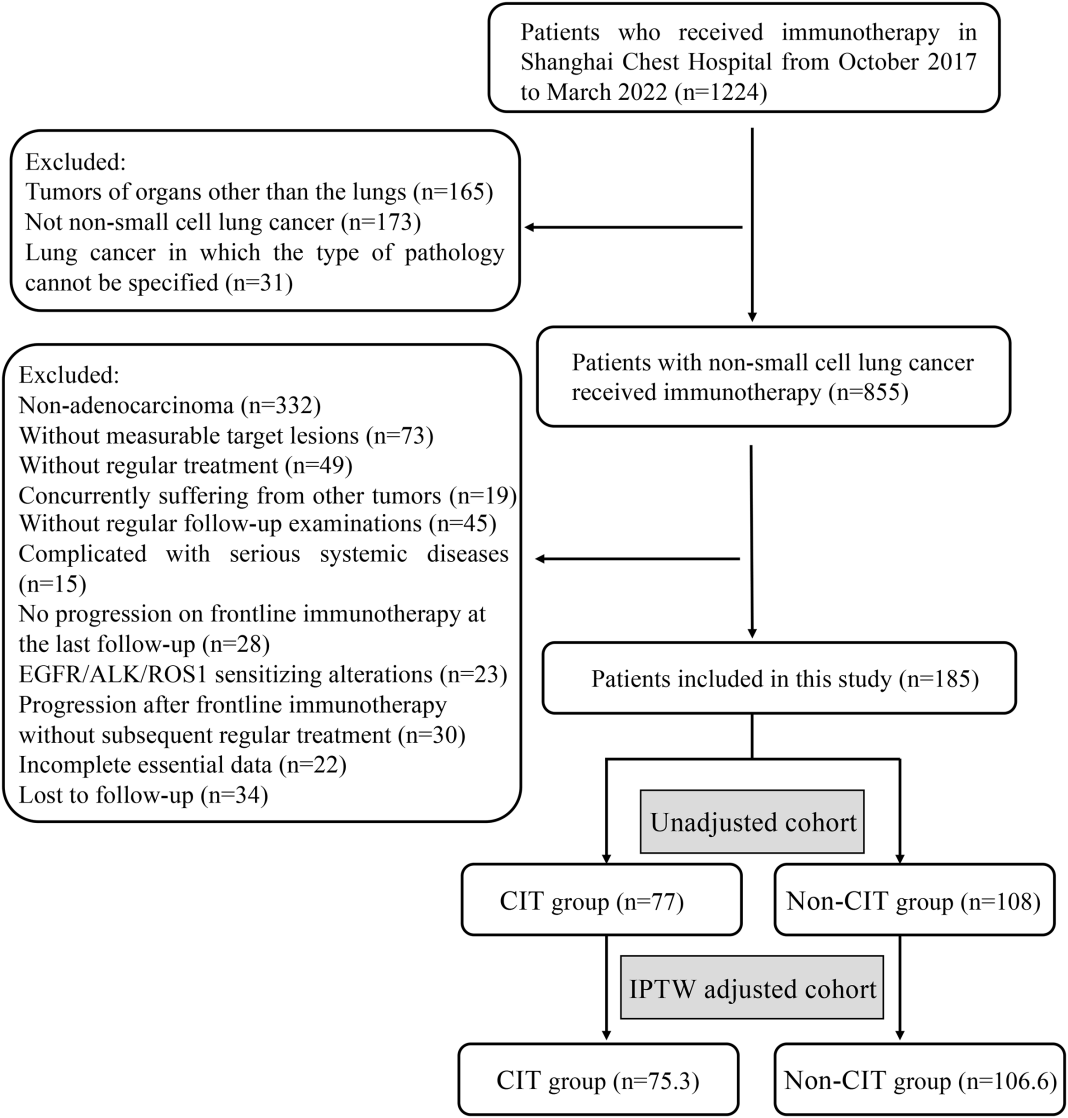
Diagram illustrating the steps for selecting patients.

**Figure 2:**
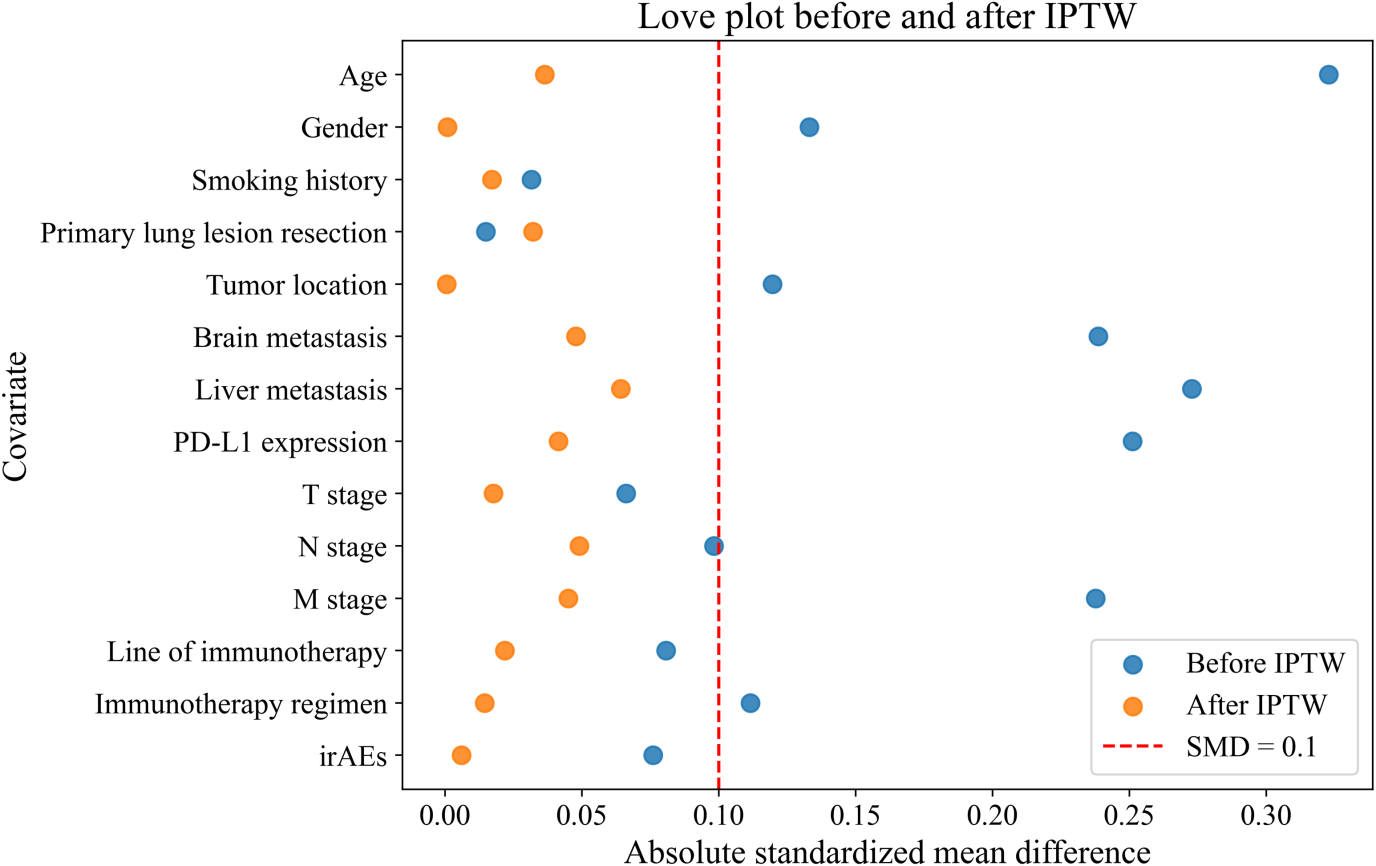
Love plot showing SMDs for baseline covariates between the cross-line immunotherapy (CIT) and Non-CIT groups before and after IPTW. After IPTW adjustment, covariate balance was substantially improved, with all absolute SMDs below 0.1. SMD, standardized mean difference; IPTW, inverse probability of treatment weighting; PD-L1, programmed death receptor ligand 1; irAEs, immune-related adverse events.

A total of 185 patients with histologically confirmed unresectable lung adenocarcinoma were included, comprising 77 (41.6%) in the CIT group and 108 (58.4%) in the Non-CIT group. The median age was 63 years (range, 35–84), and 143 (77.3%) were male. A total of 56.8% of patients were current or former smokers. All enrolled patients had an ECOG performance status score ≤2. The majority were diagnosed at stage IV (84.3%). Stage IV disease was eligible for inclusion, provided that the primary tumor was lung adenocarcinoma; the exclusion criterion regarding metastatic disease referred only to non-pulmonary primary tumors metastatic to the lung. All patients received ICIs–based therapy, either as monotherapy or in combination with chemotherapy or antiangiogenic agents, etc. The treatment line was defined based on the line of therapy at which immunotherapy was first administered. Among them, 110 (59.5%) received first-line immunotherapy, while 75 (40.5%) received second-line or later immunotherapy. PD-L1 expression data were available for 121 patients, of whom 79 (42.7%) had a tumor proportion score (TPS) ≥1%. The distribution of baseline clinical and pathological characteristics (including age, gender, smoking history, resection of primary lung lesions, tumor location, treatment lines, and PD-L1 expression, etc.) was well balanced between the CIT and Non-CIT groups, with no statistically significant differences (all P > 0.05). **Figure 1** outlines the patient enrollment process, while **Table 1** provides an overview of the baseline characteristics of the immunotherapy-treated cohort. After IPTW adjustment, baseline characteristics between the CIT and Non-CIT groups were well balanced, with all absolute SMDs below 0.1, as further demonstrated by the Love plot (**Figure 2**). The grouping strategy and survival definitions are summarized in **Supplementary Additional Figure 1**.

**Table 1:** Patient characteristics before and after IPTW.

Variable	Category	Before weighting				After weighting			
		CIT (n=77)	Non-CIT (n=108)	P-value	|SMD|	CIT (n=75.3)	Non-CIT (n=106.6)	P-value	|SMD|
Age (years), n (%)	<60	34 (44.2%)	38 (35.2%)	0.217	0.323	27.9 (37.0%)	40.5 (38.0%)	0.897	0.036
	≥60	43 (55.8%)	70 (64.8%)			47.4 (63.0%)	66.1 (62.0%)		
Gender, n (%)	Male	57 (74.0%)	86 (79.6%)	0.370	0.133	57.6 (76.5%)	81.6 (76.5%)	0.996	0.001
	Female	20 (26.0%)	22 (20.4%)			17.7 (23.5%)	25.0 (23.5%)		
Smoking history	Never	34 (44.2%)	46 (42.6%)	0.833	0.032	33.1 (44.0%)	47.8 (44.8%)	0.910	0.017
	Current/former	43 (55.8%)	62 (57.4%)			42.2 (56.0%)	58.8 (55.2%)		
Primary lung lesion resection	No	54 (70.1%)	75 (69.4%)	0.920	0.015	54.5 (72.3%)	75.5 (70.9%)	0.831	0.032
	Yes	23 (29.9%)	33 (30.6%)			20.9 (27.7%)	31.1 (29.1%)		
Tumor location	Central	18 (23.4%)	20 (18.5%)	0.420	0.120	16.2 (21.5%)	22.9 (21.5%)	0.997	0.001
	Peripheral	59 (76.6%)	88 (81.5%)			59.2 (78.5%)	83.7 (78.5%)		
Brain metastasis	No	55 (71.4%)	65 (60.2%)	0.114	0.239	50.6 (67.1%)	69.2 (64.9%)	0.751	0.048
	Yes	22 (28.6%)	43 (39.8%)			24.8 (32.9%)	37.4 (35.1%)		
Liver metastasis	No	73 (94.8%)	94 (87.0%)	0.079	0.273	68.9 (91.5%)	95.5 (89.6%)	0.672	0.064
	Yes	4 (5.2%)	14 (13.0%)			6.4 (8.5%)	11.1 (10.4%)		
PD-L1 expression	TPS<1%	15 (19.5%)	27 (25.0%)	0.236	0.251	16.3 (21.7%)	24.9 (23.4%)	0.956	0.041
	TPS≥1%	30 (39.0%)	49 (45.4%)			32.8 (43.5%)	46.2 (43.3%)		
	Unknown	32 (41.6%)	32 (29.6%)			26.3 (34.8%)	35.5 (33.3%)		
T stage	T0-2	46 (59.7%)	68 (63.0%)	0.657	0.066	46.8 (62.1%)	67.1 (63.0%)	0.907	0.018
	T3-4	31 (40.3%)	40 (37.0%)			28.5 (37.9%)	39.5 (37.0%)		
N stage	N0-1	15 (19.5%)	17 (15.7%)	0.507	0.098	13.7 (18.1%)	17.3 (16.3%)	0.744	0.049
	N2-3	62 (80.5%)	91 (84.3%)			61.7 (81.9%)	89.3 (83.7%)		
M stage	M0	16 (20.8%)	13 (12.0%)	0.107	0.238	13.3 (17.7%)	17.1 (16.0%)	0.764	0.045
	M1	61 (79.2%)	95 (88.0%)			62.0 (82.3%)	89.5 (84.0%)		
Line of immunotherapy ^a^	First-line	44 (57.1%)	66 (61.1%)	0.558	0.081	44.3 (58.7%)	63.8 (59.8%)	0.884	0.022
	Second-line or later	33 (42.9%)	42 (38.9%)			31.1 (41.3%)	42.8 (40.2%)		
Immunotherapy regimen ^b^	Combination therapy	58 (75.3%)	76 (70.4%)	0.457	0.112	53.9 (71.5%)	75.6 (70.9%)	0.923	0.015
	Monotherapy	19 (24.7%)	32 (29.6%)			21.4 (28.5%)	31.0 (29.1%)		
irAEs ^c^	No	40 (51.9%)	52 (48.1%)	0.610	0.076	38.2 (50.7%)	53.8 (50.4%)	0.968	0.006
	Yes	37 (48.1%)	56 (51.9%)			37.1 (49.3%)	52.8 (49.6%)		

IPTW, inverse probability of treatment weighting; CIT, with cross-line immunotherapy; Non-CIT, without cross-line immunotherapy; PD-L1, programmed cell death-ligand 1; TPS, tumor proportion score; T stage, tumor stage; N stage, lymph node stage; M stage, distant metastasis stage; irAEs, immune-related adverse events.

^a^The treatment line at which immune checkpoint inhibitors were first administered. ^b^The treatment modality at the time of first immunotherapy administration (monotherapy or combination therapy). ^c^Adverse events were considered immune-related when a potential immune-mediated mechanism was suspected and alternative etiologies, such as disease progression, infection, or toxicity attributable to concomitant therapies (e.g., chemotherapy or anti-angiogenic agents), were reasonably excluded. For patients receiving combination therapy, events were reviewed by at least two independent investigators. Only adverse events judged to be probably or definitely related to immune checkpoint inhibitors were classified as irAE.

### 3.2 Efficacy outcomes

#### 3.2.1 Univariate survival analysis

**Figure 3:**
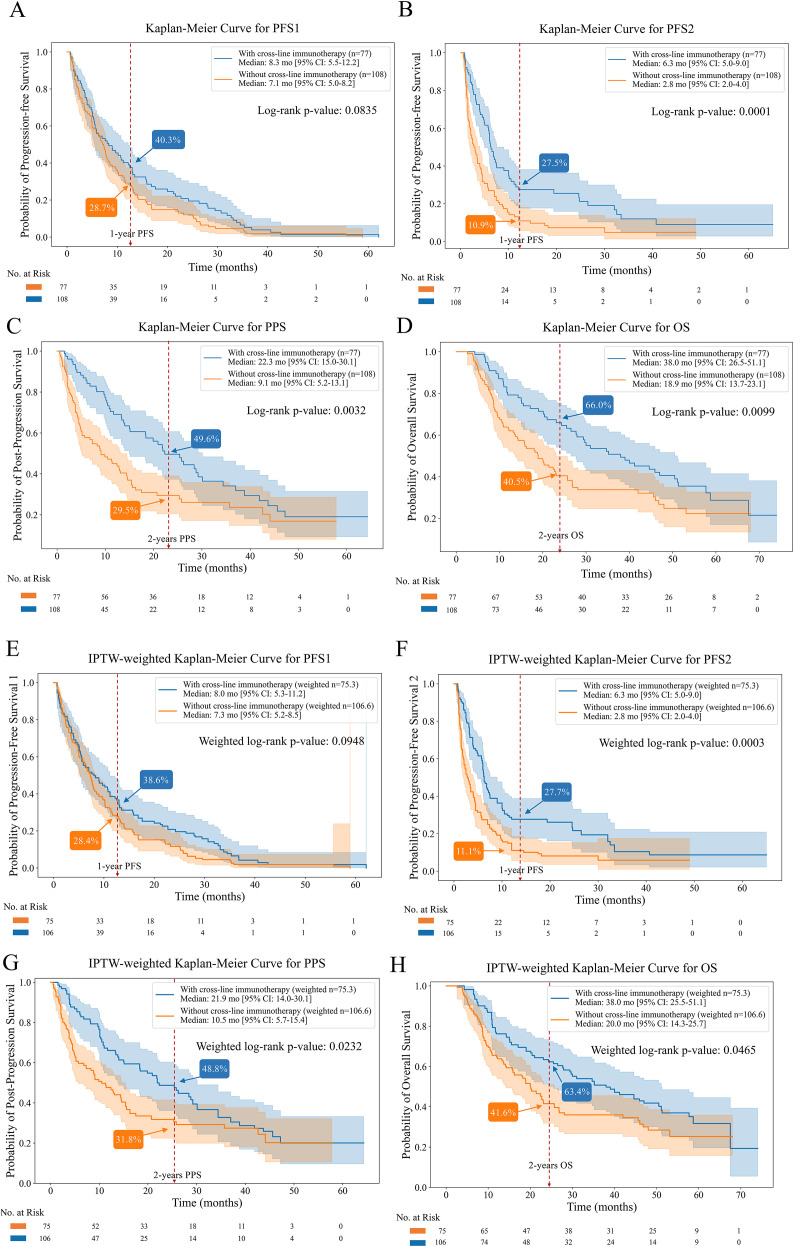
Univariate survival analysis comparing cross-line immunotherapy (CIT) and without cross-line immunotherapy (non-CIT) groups for PFS1, PFS2, PPS, and OS before and after IPTW. Kaplan–Meier survival curves for PFS1, PFS2, PPS, and OS comparing patients with and without CIT before **(A–D)** and after IPTW adjustment **(E–H)**. The log-rank test was used to evaluate differences between the two groups. CIT, cross-line immunotherapy; IPTW, inverse probability of treatment weighting; PFS, progression-free survival; PPS, post-progression survival; OS, overall survival; CI, confidence interval.

Both unweighted and IPTW-weighted Kaplan–Meier analyses were performed for PFS1, PFS2, PPS, and OS, and the corresponding survival curves are shown in **Figure 3**. In univariate analyses, there was no significant difference in PFS1 between the CIT and Non-CIT groups (median PFS1: 8.3 vs. 7.1 months; P = 0.084; **Figure 3A**), and this finding remained consistent after IPTW adjustment (median PFS1: 8.0 vs. 7.3 months; P = 0.095; **Figure 3E**). However, PFS2 was significantly longer in the CIT group compared with the Non-CIT group in the unweighted analysis (median PFS2: 6.3 vs. 2.8 months; P<0.001; **Figure 3B**), with a similar result observed after IPTW adjustment (median PFS2: 6.3 vs. 2.8 months; P<0.001; **Figure 3F**). The absence of difference in PFS1 but the significant improvement in PFS2 after IPTW adjustment suggests that the observed survival difference may be primarily associated with treatment after progression rather than initial disease control. Similarly, the combined PFS1+PFS2 analysis demonstrated significantly prolonged survival in the CIT group relative to the Non-CIT group both before and after IPTW adjustment (unweighted median PFS1+PFS2: 22.2 vs. 10.4 months; P = 0.002; IPTW-weighted median PFS1+PFS2: 20.8 vs. 10.4 months; P = 0.006; **Supplementary Additional Figure 2**).

In addition to CIT, several clinicopathologic variables were significantly associated with survival outcomes in univariate analyses. For PFS2, significant prognostic factors included PD-L1 expression (TPS≥1% vs. TPS<1%: HR = 0.59, 95%CI: 0.40–0.88, P = 0.009; Unknown vs. TPS<1%: HR = 0.61, 95%CI: 0.40–0.92, P = 0.017), M stage (M0 vs. M1: HR = 0.57, 95%CI: 0.36–0.91, P = 0.017), and the presence of irAEs (Yes vs. No: HR = 0.67, 95%CI: 0.48–0.92, P = 0.013). Meanwhile, the IPTW-adjusted univariate results for PFS2 were broadly consistent with the corresponding unweighted analyses, supporting the robustness of the observed association between CIT and improved PFS2. While the results of unweighted and IPTW-adjusted univariate analyses for PFS2 are presented in **Table 2**. For combined PFS1+PFS2, significant factors included gender (Male vs. Female: HR = 0.66, 95%CI: 0.46–0.95, P = 0.026), smoking history (Current/former vs. Never: HR = 0.68, 95%CI: 0.49–0.93, P = 0.015), liver metastasis (Yes vs. No: HR = 1.70, 95%CI: 1.02–2.82, P = 0.041), PD-L1 expression (TPS≥1% vs. TPS<1%: HR = 0.49, 95%CI: 0.33–0.72, P<0.001; Unknown vs. TPS <1%: HR = 0.52, 95%CI: 0.35–0.79, P = 0.002), M stage (M0 vs. M1: HR = 0.58, 95%CI: 0.37–0.92, P = 0.021), immunotherapy regimen (Combination therapy vs. Monotherapy: HR = 0.67, 95%CI: 0.48–0.95, P = 0.025), and irAEs (Yes vs. No: HR = 0.58, 95%CI: 0.42–0.79, P = 0.001). The IPTW-adjusted univariate analyses for PFS1+PFS2 similarly identified multiple significant prognostic factors, and the results were generally consistent with the corresponding unweighted analyses (**Supplementary Additional Table 2**).

**Table 2:** Univariate Analyses of clinical parameters on PFS outcomes before and after IPTW.

	Before weighting (PFS2)			After weighting (PFS2)		
Variable	Median survival (95% CI)	HR (95% CI)	*P*	Median survival (95% CI)	HR (95% CI)	*P*
Cross-line immunotherapy						
CIT	6.3 (5.0-9.0)	0.52 (0.38-0.72)	<0.001	6.3 (5.0-9.0)	0.54 (0.39-0.77)	<0.001
Non-CIT	2.8 (2.0-4.0)	1.0 (Reference)	–	2.8 (2.0-4.0)	1.0 (Reference)	–
Age						
<60	4.5 (2.8-5.7)	1.03 (0.75-1.43)	0.836	4.1 (2.5-5.0)	1.00 (0.98-1.01)	0.594
≥60	4.1 (3.0-6.2)	1.0 (Reference)	–	4.3 (3.2-6.3)	1.0 (Reference)	–
Gender						
Male	4.3 (3.1-5.6)	0.77 (0.53-1.10)	0.145	4.3 (3.2-5.6)	0.75 (0.54-1.04)	0.081
Female	4.1 (2.0-6.0)	1.0 (Reference)	–	4.1 (1.8-6.3)	1.0 (Reference)	–
Smoking history						
Current/former	4.5 (3.1-6.0)	0.77 (0.56-1.05)	0.098	4.3 (3.2-5.6)	0.80 (0.58-1.11)	0.177
Never	4.0 (2.3-5.8)	1.0 (Reference)	–	4.1 (2.7-6.0)	1.0 (Reference)	–
Primary lung lesion resection						
Yes	4.5 (2.5-6.8)	0.98 (0.69-1.37)	0.885	4.5 (2.3-6.8)	0.98 (0.68-1.43)	0.915
No	4.1 (3.1-5.7)	1.0 (Reference)	–	4.1 (3.3-5.7)	1.0 (Reference)	–
Tumor location						
Peripheral	4.4 (3.2-5.7)	0.90 (0.62-1.31)	0.574	4.3 (3.2-5.7)	0.95 (0.66-1.36)	0.776
Central	4.0 (2.5-7.4)	1.0 (Reference)	–	4.1 (2.6-7.8)	1.0 (Reference)	–
Brain Metastasis						
Yes	3.9 (2.5-5.6)	1.07 (0.77-1.48)	0.679	4.4 (3.1-6.3)	0.93 (0.66-1.31)	0.683
No	4.5 (3.1-6.0)	1.0 (Reference)	–	4.1 (3.0-5.8)	1.0 (Reference)	–
Liver Metastasis						
Yes	3.2 (1.5-6.8)	1.43 (0.86-2.38)	0.164	3.2 (1.5-6.8)	1.45 (0.98-2.13)	0.062
No	4.4 (3.4-5.7)	1.0 (Reference)	–	4.4 (3.4-5.7)	1.0 (Reference)	–
PD-L1 expression						
TPS<1%	3.2 (1.6-4.5)	1.0 (Reference)	–	3.2 (1.8-4.8)	1.0 (Reference)	–
TPS≥1%	4.5 (3.1-7.0)	0.59 (0.40-0.88)	0.009	4.5 (3.2-7.2)	0.57 (0.40-0.84)	0.004
Unknown	4.5 (2.1-6.5)	0.61 (0.40-0.92)	0.017	4.1 (2.1-6.3)	0.64 (0.43-0.97)	0.033
T Stage						
T0-2	5.7 (3.4-6.2)	0.82 (0.59-1.13)	0.219	5.7 (3.3-6.8)	0.81 (0.58-1.15)	0.234
T3-4	3.5 (2.3-4.4)	1.0 (Reference)	–	3.9 (2.5-4.4)	1.0 (Reference)	–
N stage						
N0-1	4.9 (2.3-7.0)	0.88 (0.58-1.32)	0.522	4.9 (2.3-7.0)	0.88 (0.57-1.37)	0.567
N2-3	4.0 (3.1-5.6)	1.0 (Reference)	–	4.1 (3.2-5.6)	1.0 (Reference)	–
M stage						
M0	7.5 (4.8-11.1)	0.57 (0.36-0.91)	0.017	8.9 (5.6-11.5)	0.60 (0.40-0.89)	0.011
M1	3.9 (2.8-4.5)	1.0 (Reference)	–	3.9 (2.8-4.5)	1.0 (Reference)	–
Line of immunotherapy						
First-line	5.0 (3.2-6.2)	0.88 (0.64-1.21)	0.443	5.0 (3.2-6.2)	0.86 (0.61-1.19)	0.361
Second-line or later	3.9 (2.3-4.5)	1.0 (Reference)	–	3.9 (2.3-4.5)	1.0 (Reference)	–
Immunotherapy regimen						
Combination therapy	4.9 (3.9-6.0)	0.73 (0.52-1.03)	0.077	4.9 (3.9-6.2)	0.70 (0.47-1.03)	0.070
Monotherapy	2.6 (1.5-4.1)	1.0 (Reference)	–	2.5 (1.5-4.0)	1.0 (Reference)	–
irAEs						
Yes	5.7 (3.5-6.8)	0.67 (0.48-0.92)	0.013	5.7 (3.5-6.8)	0.70 (0.50-0.97)	0.033
No	3.2 (2.3-4.4)	1.0 (Reference)	–	3.4 (2.5-4.5)	1.0 (Reference)	–

IPTW, inverse probability of treatment weighting; PFS, progression-free survival; CI, confidence interval; HR, Hazard Ratio; CIT, with cross-line immunotherapy; Non-CIT, without cross-line immunotherapy; PD-L1, programmed cell death-ligand 1; TPS, tumor proportion score; T stage, tumor stage; N stage, lymph node stage; M stage, distant metastasis stage; irAEs, immune-related adverse events.

Furthermore, univariate analysis of PPS and OS revealed that patients in the CIT group experienced significantly longer survival than those in the Non-CIT group (median PPS: 22.3 vs. 9.1 months, P = 0.003; median OS: 38.0 vs. 18.9 months, P = 0.010; **Figures 3C, D**). These associations remained consistent after IPTW adjustment (median PPS: 21.9 vs. 10.5 months, P = 0.023; median OS: 38.0 vs. 20.0 months, P = 0.047; **Figures 3G, H**). These results indicate that continued immunotherapy after progression is associated with improved post-progression and overall survival outcomes.

In addition to CIT, several other clinical factors were also associated with survival outcomes in univariate analyses. For IPTW-adjusted PPS, M stage (M0 vs. M1: HR = 0.56, 95%CI: 0.31–1.02, P = 0.029) and the occurrence of irAEs (Yes vs. No: HR = 0.62, 95%CI: 0.42–0.93, P = 0.010). was significantly correlated with prolonged survival. For IPTW-adjusted OS, significant predictors included M stage (M0 vs. M1: HR = 0.52, 95%CI: 0.30–0.90, P = 0.013), immunotherapy regimen (Combination therapy vs. Monotherapy: HR = 0.66, 95%CI: 0.44–0.97, P = 0.027), and the presence of irAEs (Yes vs. No: HR = 0.55, 95%CI: 0.37–0.83, P = 0.001). The unadjusted univariate results for PPS and OS were broadly consistent with the corresponding IPTW-adjusted analyses, supporting the robustness of the observed associations. The IPTW-adjusted univariate analyses for PPS and OS are shown in **Table 3**, while the corresponding unweighted PPS and OS analyses are provided in **Supplementary Additional Table 3**.

**Table 3:** Univariate analyses of clinical parameters on PPS and OS outcomes after IPTW.

	PPS			OS		
Variable	Median survival (95% CI)	HR (95% CI)	*P*	Median survival (95% CI)	HR (95% CI)	*P*
Cross-line immunotherapy						
CIT	21.9 (14.0-30.1)	0.66 (0.44-0.97)	0.023	38.0 (25.5-51.1)	0.69 (0.46-1.02)	0.047
Non-CIT	10.5 (5.7-15.4)	1.0 (Reference)	–	20.0 (14.3-25.7)	1.0 (Reference)	–
Age						
<60	16.3 (11.4-30.1)	0.85 (0.59-1.25)	0.402	31.0 (18.2-47.0)	0.95 (0.65-1.39)	0.776
≥60	13.1 (9.7-19.9)	1.0 (Reference)	–	23.0 (18.8-28.1)	1.0 (Reference)	–
Gender						
Male	11.4 (9.1-15.4)	1.36 (0.86-2.15)	0.155	20.4 (15.8-35.2)	1.16 (0.74-1.83)	0.500
Female	21.8 (15.4-37.0)	1.0 (Reference)	–	31.0 (23.1-46.0)	1.0 (Reference)	–
Smoking history						
Current/former	10.5 (8.9-14.4)	1.22 (0.83-1.80)	0.283	20.3 (15.7-35.2)	1.05 (0.71-1.54)	0.800
Never	20.7 (14.5-28.4)	1.0 (Reference)	–	27.9 (22.4-42.0)	1.0 (Reference)	–
Primary lung lesion resection						
Yes	19.9 (10.5-28.4)	0.96 (0.66-1.40)	0.860	29.4 (19.0-48.0)	1.00 (0.68-1.47)	0.996
No	13.8 (10.1-16.3)		–	22.7 (16.2-35.2)	1.0 (Reference)	–
Tumor location						
Peripheral	15.0 (10.5-20.9)	1.20 (0.70-2.07)	0.422	25.5 (19.0-39.0)	1.16 (0.69-1.95)	0.996
Central	13.8 (6.0-44.1)	1.0 (Reference)	–	26.5 (15.0-53.1)	1.0 (Reference)	–
Brain Metastasis						
Yes	11.4 (7.3-30.1)	0.91 (0.59-1.39)	0.628	21.4 (14.3-46.8)	0.92 (0.60-1.41)	0.676
No	15.4 (11.0-21.8)	1.0 (Reference)	–	25.7 (20.1-39.0)	1.0 (Reference)	–
Liver Metastasis						
Yes	10.1 (2.5-25.8)	1.41 (0.80-2.47)	0.253	15.0 (7.4-inf)	1.54 (0.82-2.88)	0.150
No	15.1 (11.3-21.8)	1.0 (Reference)	–	25.7 (20.1-39.0)	1.0 (Reference)	–
PD-L1 expression						
TPS<1%	10.1 (4.9-29.2)	1.0 (Reference)	–	20.3 (9.4-44.6)	1.0 (Reference)	–
TPS≥1%	15.4 (10.5-22.3)	0.87 (0.53-1.43)	0.571	26.8 (18.9-45.6)	0.72 (0.44-1.19)	0.150
Unknown	18.4 (9.7-28.4)	0.83 (0.48-1.45)	0.452	25.5 (20.1-47.0)	0.73 (0.42-1.24)	0.232
T Stage						
T0-2	15.4 (10.8-25.8)	0.80 (0.55-1.17)	0.231	29.7 (21.0-46.8)	0.75 (0.51-1.10)	0.117
T3-4	12.8 (8.1-16.3)	1.0 (Reference)	–	20.1 (15.0-26.5)	1.0 (Reference)	–
N stage						
N0-1	21.8 (9.9-inf)	0.68 (0.40-1.14)	0.128	42.0 (18.8-inf)	0.75 (0.45-1.26)	0.275
N2-3	13.8 (10.1-18.3)	1.0 (Reference)	–	23.1 (18.9-31.0)	1.0 (Reference)	–
M stage						
M0	34.4 (11.3-inf)	0.56 (0.31-1.02)	0.029	38.0 (22.7-inf)	0.52 (0.30-0.90)	0.013
M1	13.3 (10.1-17.5)	1.0 (Reference)	–	21.4 (16.2-27.9)	1.0 (Reference)	–
Line of Immunotherapy						
First-line	14.0 (10.1-19.9)	0.97 (0.66-1.42)	0.861	25.7 (18.9-44.6)	0.89 (0.61-1.29)	0.507
Second-line or later	16.3 (9.7-26.2)	1.0 (Reference)	–	23.1 (17.1-35.2)	1.0 (Reference)	–
Immunotherapy regimen						
Combination therapy	15.4 (12.4-22.3)	0.73 (0.49-1.08)	0.100	27.9 (20.4-44.6)	0.66 (0.44-0.97)	0.027
Monotherapy	11.0 (5.2-20.7)	1.0 (Reference)	–	18.9 (12.0-26.8)	1.0 (Reference)	–
irAEs						
Yes	25.3 (13.8-37.0)	0.62 (0.42-0.93)	0.010	45.6 (26.9-53.1)	0.55 (0.37-0.83)	0.001
No	10.5 (8.2-14.5)	1.0 (Reference)	–	15.9 (11.0-21.4)	1.0 (Reference)	–

IPTW, inverse probability of treatment weighting; PPS, post-progression survival; OS, overall survival; CI, confidence interval; HR, Hazard Ratio; CIT, with cross-line immunotherapy; Non-CIT, without cross-line immunotherapy; PD-L1, programmed cell death-ligand 1; TPS, tumor proportion score; T stage, tumor stage; N stage, lymph node stage; M stage, distant metastasis stage; irAEs, immune-related adverse events.

#### 3.2.2 Multivariate survival analysis

To further determine independent prognostic factors, variables with significance in univariate analysis, together with some important clinically parameters, were entered into multivariable Cox proportional hazards models, including CIT, gender, smoking history, liver metastasis, PD-L1 expression, M stage, immunotherapy regimen, and irAEs. The detailed IPTW-adjusted multivariable results for all endpoints are summarized in **Table 4**, whereas the corresponding unadjusted multivariable analyses are provided in **Supplementary Additional Table 4**. In the unadjusted multivariable models, CIT was also significantly associated with improved PFS2, PFS1+PFS2, PPS, and OS, and the direction and magnitude of these associations were broadly consistent with the IPTW-adjusted results. Briefly, the key findings for each endpoint are as follows.

**Table 4:** Multivariate analyses of clinical parameters on PFS and OS outcomes after IPTW.

	PFS2		PFS1+PFS2		PPS		OS	
Variable	HR (95%CI)	*P*	HR (95%CI)	*P*	HR (95%CI)	*P*	HR (95%CI)	*P*
Cross-line immunotherapy								
CIT vs. Non-CIT	0.57 (0.40-0.80)	0.001	0.59 (0.41-0.86)	0.006	0.64 (0.45-0.92)	0.017	0.64 (0.43-0.94)	0.024
Gender								
Male vs. Female	0.82 (0.49-1.35)	0.430	0.92 (0.54-1.58)	0.762	1.73 (0.98-3.04)	0.057	1.74 (0.96-3.15)	0.070
Smoking history								
Current/former vs. Never	0.96 (0.60-1.55)	0.878	0.77 (0.48-1.23)	0.269	1.00 (0.61-1.62)	0.986	0.87 (0.52-1.44)	0.590
Liver Metastasis								
Yes vs. No	1.10 (0.68-1.80)	0.689	1.45 (0.82-2.56)	0.203	1.48 (0.85-2.59)	0.169	1.59 (0.88-2.89)	0.125
PD-L1 expression								
TPS≥1% vs. TPS<1%	0.60 (0.40-0.90)	0.014	0.52 (0.35-0.78)	<0.001	0.90 (0.56-1.46)	0.681	0.73 (0.46-1.17)	0.195
Unknown vs. TPS<1%	0.52 (0.34-0.80)	0.003	0.46 (0.31-0.70)	0.001	0.66 (0.39-1.13)	0.133	0.55 (0.33-0.91)	0.021
M stage								
M0 vs. M1	0.50 (0.32-0.78)	0.002	0.52 (0.34-0.79)	0.002	0.52 (0.28-0.94)	0.030	0.47 (0.27-0.83)	0.010
Immunotherapy regimen								
Combination therapy vs. Monotherapy	0.56 (0.38-0.82)	0.003	0.47 (0.31-0.70)	<0.001	0.71 (0.47-1.07)	0.099	0.57 (0.38-0.87)	0.008
irAEs								
Yes vs. No	0.67 (0.47-0.96)	0.029	0.53 (0.36-0.77)	<0.001	0.52 (0.34-0.78)	0.002	0.44 (0.29-0.67)	<0.001

IPTW, inverse probability of treatment weighting; PFS, progression-free survival; PPS, post-progression survival; OS, overall survival; CI, confidence interval; HR, Hazard Ratio; CIT, with cross-line immunotherapy; Non-CIT, without cross-line immunotherapy; PD-L1, programmed cell death-ligand 1; TPS, tumor proportion score; M stage, distant metastasis stage; irAEs, immune-related adverse events.

In the IPTW-adjusted multivariable analysis of PFS2, five variables emerged as independent prognostic significance. Patients who received CIT had significantly prolonged PFS2 compared with those who did not (HR = 0.57, 95%CI 0.40–0.80; P = 0.001). Similarly, PD-L1 expression remained an important predictor of better outcomes, with both TPS ≥ 1% (HR = 0.60, 95%CI 0.40–0.90; P = 0.014) and unknown PD-L1 status (HR = 0.52, 95%CI 0.34–0.80; P = 0.003) associated with improved PFS2. In addition, patients with M0 disease experienced significantly longer PFS2 (HR = 0.50, 95%CI 0.32–0.78; P = 0.002) than M1, and those receiving combination immunotherapy showed superior survival compared with monotherapy (HR = 0.56, 95%CI 0.38–0.82; P = 0.003). The occurrence of irAEs also independently predicted favorable PFS2 (HR = 0.67, 95%CI 0.47–0.96; P = 0.029), further linking immune activation to durable clinical benefit. The IPTW-adjusted analysis for the composite endpoint PFS1+PFS2 yielded consistent findings. The independent predictors for a longer PFS1+PFS2 were: receiving CIT (HR = 0.59, 95%CI: 0.41-0.86; P = 0.006), PD-L1 expression (TPS ≥1%: HR = 0.52, 95%CI: 0.35-0.78, P<0.001; Unknown: HR = 0.46, 95%CI: 0.31-0.70, P = 0.001), M0 stage (HR = 0.52, 95%CI: 0.34-0.79; P = 0.002), combination immunotherapy regimen (HR = 0.47, 95%CI: 0.31-0.70; P<0.001), and the development of irAEs (HR = 0.53, 95%CI: 0.36-0.77; P<0.001).

For PPS, the IPTW-adjusted multivariable analysis identified three independent factors. CIT (HR = 0.64, 95%CI: 0.45-0.92; P = 0.017), M0 stage (HR = 0.52, 95%CI: 0.28-0.94; P = 0.030), and the occurrence of irAEs (HR = 0.52, 95%CI: 0.34-0.78; P = 0.002) were all significantly associated with improved survival after disease progression. Finally, in the IPTW-adjusted multivariable analysis of OS, five factors retained independent significance. Favorable OS was independently associated with receiving CIT (HR = 0.64, 95%CI: 0.43-0.94; P = 0.024), an unknown PD-L1 status compared to TPS <1% (HR = 0.55, 95%CI: 0.33-0.91; P = 0.021), M0 stage (HR = 0.47, 95%CI: 0.27-0.83; P = 0.010), combination immunotherapy (HR = 0.57, 95%CI: 0.38-0.87; P = 0.008), and the development of irAEs (HR = 0.44, 95% CI: 0.29-0.67; P<0.001).

#### 3.2.3 Subgroup analysis

To further explore the consistency of the survival association with CIT across different patient populations and to identify the subpopulations that may derive greater benefit, subgroup analyses were performed for PFS2, PFS1+PFS2, PPS, and OS using Cox proportional hazards models. Subgroup analyses were conducted under the IPTW framework, and HRs with 95%CIs were estimated and visually summarized using forest plots. Given the limited sample size within some subgroups, these analyses were considered exploratory and were not powered for definitive subgroup comparisons.

**Figure 4:**
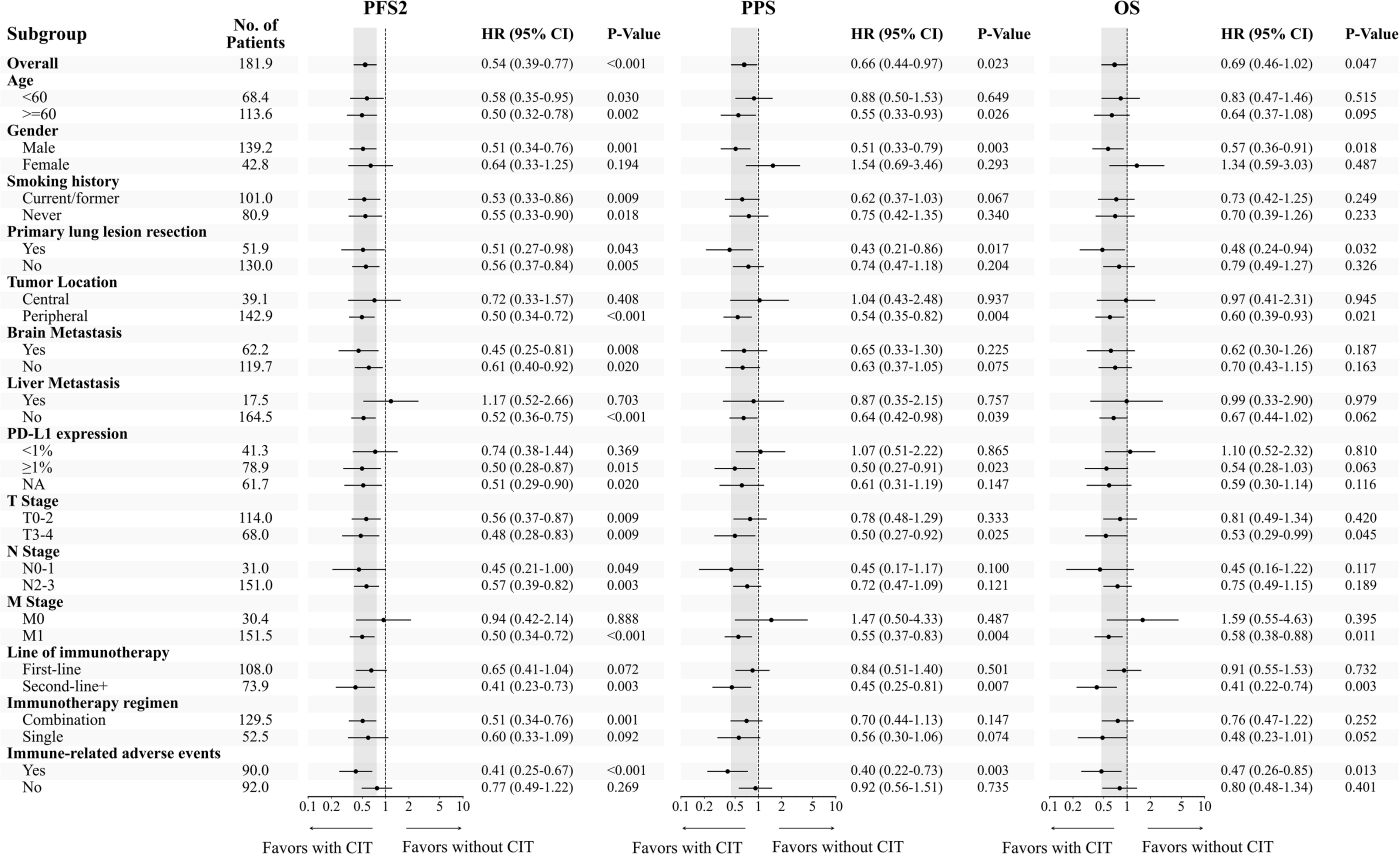
Subgroup analysis of cross-line immunotherapy (CIT) for PFS2, PPS and OS after IPTW adjustment. Forest plot illustrating the hazard ratios (HRs) and 95% confidence intervals (CIs) of cross-line immunotherapy (CIT) versus non-CIT across different clinical subgroups for PFS2, PPS and OS after IPTW adjustment. HRs were calculated using a Cox proportional hazards regression model. The vertical dashed line indicates the reference HR of 1, and the horizontal lines represent 95% CIs. IPTW, inverse probability of treatment weighting; CIT, cross-line immunotherapy; HR, hazard ratios; CI, confidence interval; PD-L1, programmed death receptor ligand 1; T stage, tumor stage; N stage, lymph node stage; M stage, distant metastasis stage.

In the IPTW-adjusted subgroup analysis of PFS2, CIT was associated with a statistically significant benefit in most subgroups (**Figure 4**). Notably, the association appeared more evident in subgroups such as patients aged ≥60 years, males, those with a current or former smoking history, without primary lung lesion resection, peripheral tumors, presence of brain metastases, absence of liver metastases, N2–3, M1 stage, those receiving second-line or later immunotherapy, combination therapy, and those who developed irAEs. However, these subgroup findings should be interpreted cautiously, as they may reflect differences in underlying tumor-immune biology, such as PD-L1 expression, smoking-associated tumor mutational burden, or other unmeasured biomarkers, rather than true effect modification by age or smoking status. Conversely, no statistically significant association with improved PFS2 was observed in subgroups including female patients, those with central tumor location, presence of liver metastases, PD-L1 TPS <1%, M0 stage, or without irAEs. The unadjusted and IPTW-adjusted subgroup analyses for PFS1+PFS2 showed a generally similar pattern, and the corresponding forest plot is provided in **Supplementary Additional Figure 3**. Similarly, for PPS, CIT demonstrated an association with longer survival in several predefined subgroups after IPTW adjustment (**Figure 4**). Patients who appeared to derive greater PPS benefit from CIT including males, peripheral tumor location, M1 stage, those receiving second-line or later immunotherapy and those who developed irAEs. However, no significant differences were detected in younger patients (<60 years), females, never-smokers, without primary lung lesion resection, central-type tumors, or those with brain or liver metastases, PD-L1 TPS <1%, T0–2 stage, M0 stage, first-line immunotherapy, or without irAEs. The IPTW-adjusted subgroup analysis for OS were generally consistent with that of PPS (**Figure 4**). The corresponding unadjusted subgroup analyses for PFS2, PPS, and OS showed generally consistent patterns and are presented in **Supplementary Additional Figure 4**. Overall, these subgroup findings should be interpreted cautiously and regarded as exploratory, given the limited sample size and event numbers in some strata. In particular, apparent associations observed in older patients and current/former smokers may reflect differences in underlying tumor-immune biology, such as PD-L1 expression, smoking-associated tumor mutational burden, or other unmeasured biomarkers, rather than true effect modification by age or smoking status.

### 3.3 Nomogram construction and validation of PPS

**Figure 5:**
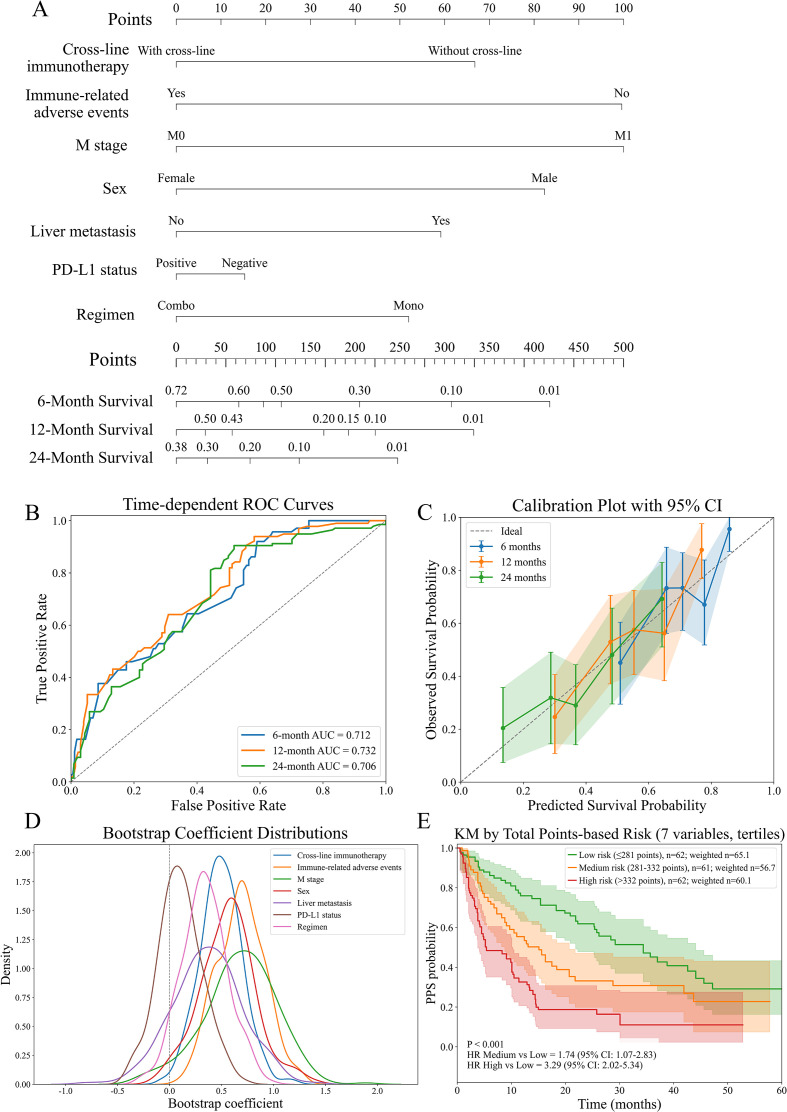
Nomogram for predicting post-progression survival (PPS) in patients with or without cross-line immunotherapy and validation of the nomogram after IPTW adjustment. **(A)** Nomogram developed based on eight independent prognostic factors identified in the multivariate Cox regression model, including cross-line immunotherapy, gender, smoking history, liver metastasis, PD-L1 expression, M stage, immunotherapy regimen, and immune-related adverse events. Each variable corresponds to a specific score on the upper scale, and the total score predicts the 6-month, 1-year, and 2-year PPS probabilities. **(B)** Time-dependent ROC curves demonstrating the predictive performance of the nomogram at 6, 12, and 18 months. **(C)** Calibration plots showing the agreement between predicted and observed PPS probabilities. **(D)** Bootstrap coefficient distributions indicating the stability of model predictors across 1,000 re-samplings. **(E)** Kaplan–Meier survival curves comparing PPS between high- and low-risk groups stratified by total points derived from the nomogram. IPTW, inverse probability of treatment weighting; PPS, post-progression survival; PD-L1, programmed death receptor ligand 1; M stage, distant metastasis stage; ROC, receiver operating characteristic curve; AUC, area under the ROC curve; CI, confidence interval; KM, Kaplan–Meier; TP, total points.

Based on the results of IPTW-weighted multivariable Cox regression analysis, a prognostic nomogram was established to predict PPS in unresectable lung adenocarcinoma patients treated with immunotherapy who subsequently experienced disease progression (**Figure 5A**). Seven variables, including CIT, gender, liver metastasis, PD-L1 expression, M stage, immunotherapy regimen, and the occurrence of irAEs, were incorporated into the model. Each variable was assigned a specific point score, and the total score corresponded to an individualized probability of 6-, 12-, and 24-month PPS. The predictive accuracy and discriminative ability of the IPTW-adjusted nomogram were evaluated. Among 500 stratified random 70/30 splits, we selected the split corresponding to the median validation C-index as the representative split. In this split, the IPTW-adjusted 7-factor PPS nomogram showed a C-index of 0.658 (95% CI: 0.598–0.724) in the training cohort and 0.623 (95% CI: 0.520–0.726) in the validation cohort, indicating moderate discriminative ability. The IPTW-adjusted time-dependent receiver operating characteristic (ROC) curves showed that the area under the curve (AUC) values for predicting 6-month, 1-year, and 2-year PPS were 0.712, 0.732, and 0.706, respectively, indicating favorable predictive accuracy (**Figure 5B**). The calibration plot (**Figure 5C**) showed that the predicted probabilities of 6-month, 1-year, and 2-year PPS were generally consistent with the observed outcomes, closely following the ideal line overall, indicating acceptable calibration. In addition, the bootstrap validation (500 resamples) showed a stable distribution of regression coefficients without apparent overfitting, further supporting the model’s robustness (**Figure 5D**).

To further evaluate the risk-stratification performance of the nomogram, patients were stratified into three risk groups based on tertiles of the total nomogram score: low (≤281), medium (281–332], and high (>332) risk. The corresponding sample sizes were n= 62, 61, and 62, respectively. IPTW-weighted Kaplan–Meier survival analysis demonstrated a clear separation among the three risk groups (log-rank P<0.001). Compared with the low-risk group, the medium- and high-risk groups exhibited significantly worse PPS survival, with HR = 1.74 (95% CI: 1.07–2.83) and HR = 3.29 (95% CI: 2.02–5.34), respectively (**Figure 5E**).

To evaluate potential multicollinearity among the variables included in the nomogram, VIF values were calculated for all seven predictor variables. The VIF analysis revealed no significant multicollinearity issues, with all variables demonstrating VIF values well below the threshold of 2.0 (**Supplementary Additional Table 5**). The highest VIF was observed for PD-L1 expression status (VIF = 1.26). All other variables showed VIF values ranging from 1.00 to 1.10, indicating minimal intercorrelation among predictors. These results support the statistical validity of including all seven variables in the final nomogram model without concerns regarding multicollinearity-induced instability.

Together, these results suggest that the IPTW-adjusted PPS nomogram, constructed based on seven key clinical and biological variables, may provide a useful exploratory tool for prognostic assessment and risk stratification in patients with unresectable lung adenocarcinoma after immunotherapy progression.

## 4 Discussion

The introduction of ICIs has transformed the therapeutic landscape of advanced NSCLC, providing durable responses and significant survival benefits in a subset of patients. In particular, for unresectable lung adenocarcinoma, both ICI monotherapy and combination regimens with chemotherapy or antiangiogenic agents have become integral components of systemic treatment (27). However, disease progression eventually occurs in most patients following initial immunotherapy, and the optimal management strategy after progression remains undefined. In this context, the concept of “CIT”—continuing or reintroducing ICIs beyond disease progression—has gained increasing clinical interest. Nevertheless, the true efficacy of CIT and its potential survival benefits across different clinical subgroups have not been thoroughly validated. Therefore, this study aims to evaluate the clinical outcomes of CIT in patients with advanced lung adenocarcinoma and to identify which populations may derive the greatest benefit.

In this retrospective cohort study of patients with unresectable lung adenocarcinoma who progressed after initial ICIs therapy, we observed that continuation of immunotherapy beyond progression CIT was associated with favorable outcomes. Our study demonstrated that PFS1 showed no difference between the CIT group and the non-CIT group, but CIT significantly prolonged PFS2 and yielded improvements in both PPS and OS. These findings persisted after adjustment for a range of clinical covariates in multivariable Cox models, underscoring a likely benefit of maintaining ICIs exposure when clinically feasible. The inclusion of different PD-1/PD-L1 inhibitors and combination regimens reflects the real-world implementation of CIT in clinical practice. Nevertheless, this treatment heterogeneity should be considered when interpreting the results, as this study was designed to evaluate the overall strategy of continuing PD-1/PD-L1 blockade beyond progression rather than to compare the efficacy of individual ICI agents. Similarly, radiotherapy represents an important but highly heterogeneous component of post-progression management, particularly for oligoprogressive disease. In this retrospective cohort, radiotherapy varied substantially in timing, site, intent, dose-fractionation, and repeated use; therefore, a simple binary classification was not considered sufficient to reliably evaluate its contribution to survival outcomes.

Several observations support a biologic and clinical rationale for this benefit. Firstly, the lack of difference in PFS1 but significant prolongation in PFS2 suggests that the benefit of CIT primarily stems from subsequent treatment phases rather than initial disease control. This pattern is biologically plausible in the context of the delayed and dynamic nature of antitumor immunity induced by PD-1/PD-L1 blockade. Unlike cytotoxic therapy, ICIs may reshape the tumor-immune equilibrium over time by reinvigorating exhausted T cells, promoting the expansion or replacement of tumor-reactive T-cell clones, and sustaining effector or memory T-cell populations (28, 29). Therefore, radiographic progression after initial ICI therapy may not necessarily indicate complete loss of immune modulation in all patients. Continued PD-1/PD-L1 blockade, particularly when combined with subsequent chemotherapy or antiangiogenic therapy, may help preserve residual immune pressure while allowing additional treatment partners to enhance antigen release, modulate the tumor microenvironment, or improve immune-cell infiltration (30, 31). In this context, the improvement in PFS2 despite similar PFS1 may reflect delayed or downstream immunological effects during subsequent treatment, although this hypothesis requires prospective translational validation. Secondly, we observed that irAEs were significantly associated with improved PFS2, PPS and OS, maintaining independent significance in multivariate models—consistent with prior studies. irAEs often reflect more active immune responses, thereby correlating with superior therapeutic outcomes. Thirdly, the repeated confirmation of M stage as an independent prognostic factor indicates that tumor burden substantially influences the treatment trajectory following immunotherapy progression. However, the apparent benefit observed in older patients and current/former smokers should be interpreted with caution. These clinical characteristics may not represent true predictive modifiers by themselves. But may instead serve as surrogate markers for underlying tumor-immune biology. For example, smoking-associated lung cancers are often characterized by higher mutational burden and neoantigen load, which may influence responsiveness to PD-1/PD-L1 blockade. Because tumor mutational burden (TMB) and other emerging biomarkers were not uniformly available in this retrospective cohort, we could not fully distinguish clinical effect modification from biomarker-driven heterogeneity. In addition, the subgroup variable of prior primary lung lesion resection referred to patients who had undergone surgery before developing unresectable recurrent or metastatic disease; all patients were unresectable at the time of ICI treatment and post-progression treatment evaluation.

To facilitate exploratory risk stratification, we developed an IPTW-adjusted nomogram incorporating clinically accessible variables to estimate PPS after immunotherapy progression. The model showed moderate discrimination and time-dependent AUCs of 0.712, 0.732 and 0.706 for 6-, 12- and 24-month PPS, respectively. Calibration plots and bootstrap resampling supported the model’s internal validity. Risk stratification by nomogram total score produced three well-separated groups. However, because external validation was not available, the nomogram should be considered hypothesis-generating rather than ready for routine clinical application. Prospective validation in independent multicenter cohorts will be required before this model can be used to guide post-progression management or follow-up strategies in clinical practice.

The superior survival outcomes observed in patients receiving CIT may be related to both tumor-intrinsic and immune-contextual factors. From a contemporary immunological perspective, the effect of continued PD-1/PD-L1 blockade should not be viewed simply as sustained T-cell activation. Tumor-reactive T cells comprise functionally heterogeneous populations, including effector-like, memory-like, progenitor-exhausted, transitory exhausted, and terminally exhausted states (32, 33). Emerging evidence suggests that responses to checkpoint blockade may depend less on globally “reversing exhaustion” and more on the presence, maintenance, and expansion of progenitor or stem-like exhausted T-cell populations capable of generating cytotoxic progeny. In contrast, terminally exhausted T cells may have limited proliferative potential and reduced responsiveness to further checkpoint inhibition (29, 33). Therefore, the benefit of CIT may depend on the residual pool of responsive tumor-reactive T cells at the time of progression.

In addition, the tumor microenvironment may strongly shape the efficacy of CIT. Immunosuppressive myeloid cells, regulatory T cells, cancer-associated fibroblasts, hypoxia, VEGF-driven abnormal vasculature, metabolic stress, and impaired antigen presentation can all contribute to immune exclusion or acquired resistance (18, 34, 35). Continued PD-1/PD-L1 blockade combined with subsequent chemotherapy or antiangiogenic therapy may, in selected patients, help preserve antitumor immune pressure while modifying antigen release, vascular permeability, and immune-cell infiltration (36). However, these mechanisms remain inferential in the present retrospective cohort because serial immune profiling was not available.

These considerations highlight the need for translational studies integrating pharmacogenomic and multi-omic approaches (17, 37, 38). Future work should incorporate tumor genomic profiling, transcriptomics, T-cell receptor sequencing, circulating tumor DNA dynamics, multiplex immunohistochemistry, single-cell and spatial immune profiling, and assessment of exhaustion-related markers such as TCF1, TOX, PD-1, TIM-3, LAG-3, and TIGIT. Such approaches may help distinguish patients with persistent, reinvigoratable antitumor immunity from those with irreversible immune escape, thereby refining biologically informed selection for CIT.

This study observed that CIT is associated with superior survival outcomes in patients with unresectable lung adenocarcinoma. This finding aligns with the recent trend of exploring the value of CIT across multiple tumor types. However, conclusions vary across different studies, warranting further analysis. More recent evidence continues to support this uncertainty. A multicenter retrospective study reported that continuation of immunotherapy beyond progression after first-line chemoimmunotherapy was associated with improved second-line PFS and OS in advanced NSCLC (39), whereas another 2024 retrospective rechallenge study found no clear benefit in an unselected NSCLC population and suggested that prior response to initial ICI therapy may be important for patient selection (40). A 2025 systematic review and pooled analysis further concluded that ICI continuation beyond radiologic progression may benefit selected patients, but emphasized that decisions should incorporate tumor biology, performance status, and emerging biomarkers (41). In addition, a *post hoc* analysis of OAK and other phase III immunotherapy cohorts suggested that inflammatory/nutritional status at progression, such as the modified Glasgow Prognostic Score, may help identify patients more likely to benefit from treatment beyond progression (42). Taken together, these studies indicate that the evidence for CIT remains heterogeneous. Some studies suggest that CIT may be associated with improved outcomes, whereas others report neutral or heterogeneous results, with potential benefit appearing to be concentrated in selected patients, such as those with prior ICI sensitivity, PD-L1-positive tumors, favorable inflammatory or nutritional status, or those receiving rational combination strategies including chemotherapy, antiangiogenic therapy, or local ablation. The majority of prior studies employed retrospective designs with small sample sizes, failing to adequately control for confounding factors and featuring limited follow-up periods. This makes it difficult to precisely define which patient populations genuinely benefit from cross-line immunotherapy. Moreover, existing analyses predominantly focus on objective response rates or PFS, frequently overlooking PPS and thus failing to comprehensively reflect post-progression efficacy. By contrast, this study offers certain advantages in research depth and data integrity. Based on reliable real-world data with extended follow-up periods and sufficient event numbers, it systematically analyzed key endpoint metrics including PFS2, PPS, and OS. The IPTW-adjusted multivariable analyses supported an independent association between CIT and improved survival outcomes, while subgroup analyses provided exploratory information on potentially responsive populations. Furthermore, we constructed and internally validated an IPTW-adjusted nomogram integrating clinically accessible variables, supplemented by risk stratification analysis, thereby providing an exploratory framework for PPS risk stratification that requires external validation before clinical application.

This study also has several limitations. First, its retrospective, single-center nature introduces potential selection bias and limits the generalizability of our findings. The consistency between the unweighted and IPTW-adjusted analyses supports the robustness of the observed association between CIT and survival outcomes. Nevertheless, IPTW can only balance measured covariates, and residual confounding from unmeasured factors may still remain because of the retrospective single-center design. In addition, nodal staging was not uniformly confirmed pathologically, as systematic EBUS/EUS-based mediastinal staging was not performed in all cases, reflecting the real-world retrospective nature of this cohort; therefore, potential misclassification of mediastinal nodal status cannot be excluded. Second, PD-L1 status was unavailable for a substantial proportion of patients, and other biomarker data such as TMB, immune infiltrates, and dynamic PD-L1 expression were not uniformly available. This incomplete biomarker information may limit the generalizability of our findings and restrict the ability to mechanistically link immune activity to CIT-associated outcomes. Third, although the IPTW-adjusted analyses supported the robustness of the main findings, subgroup analyses remained exploratory, and the nomogram was validated internally only. Because external validation was not available, the clinical applicability of the nomogram remains limited. Moreover, PPS is influenced by post-progression treatment decisions, which may reduce the independent predictive value and generalizability of the nomogram. Prospective validation in independent multicenter cohorts is required before routine clinical use, and further studies are needed to determine the optimal timing, duration, and combination partners for CIT and to clarify the biological mechanisms underlying CIT-associated outcomes through longitudinal immune profiling and tumor microenvironment assessment.

## Conclusion

In summary, our findings suggest that CIT is associated with improved survival outcomes in patients with unresectable lung adenocarcinoma after progression on prior ICI therapy, potentially through continued tumor-immune modulation and interaction with subsequent systemic treatment. CIT represents a promising treatment strategy for appropriately selected patients, while prospective multicenter studies with biomarker-informed stratification are warranted to confirm its clinical value and refine patient selection.

## Data Availability

The raw data supporting the conclusions of this article will be made available by the authors, without undue reservation.

## Supplementary material

Supplementary Additional Figure 1 Study design illustrating the grouping strategy and survival endpoints. PD, progressive disease; PFS, progression-free survival; PPS, post-progression survival; OS, overall survival.

Supplementary Additional Figure 2 Kaplan–Meier survival analyses comparing cross-line immunotherapy (CIT) and without cross-line immunotherapy (non-CIT) groups for PFS1+ PFS2 before and after IPTW adjustment. IPTW, inverse probability of treatment weighting; PFS, progression-free survival; CI, confidence interval.

Supplementary Additional Figure 3 Kaplan–Meier survival analyses comparing cross-line immunotherapy (CIT) and without cross-line immunotherapy (non-CIT) groups for PFS1+ PFS2 before and after IPTW adjustment. IPTW, inverse probability of treatment weighting; PFS, progression-free survival; CI, confidence interval.

Supplementary Additional Figure 4 Kaplan–Meier survival analyses comparing cross-line immunotherapy (CIT) and without cross-line immunotherapy (non-CIT) groups for PFS1+ PFS2 before and after IPTW adjustment. IPTW, inverse probability of treatment weighting; PFS, progression-free survival; CI, confidence interval.
