# Supplementary figures and images for: Prognostic significance of continuing immunotherapy beyond progression in unresectable lung adenocarcinoma
Source: Front Immunol. 2026 Jul 8;17:1805604. doi: 10.3389/fimmu.2026.1805604 (PMC13388323; doi:10.3389/fimmu.2026.1805604)

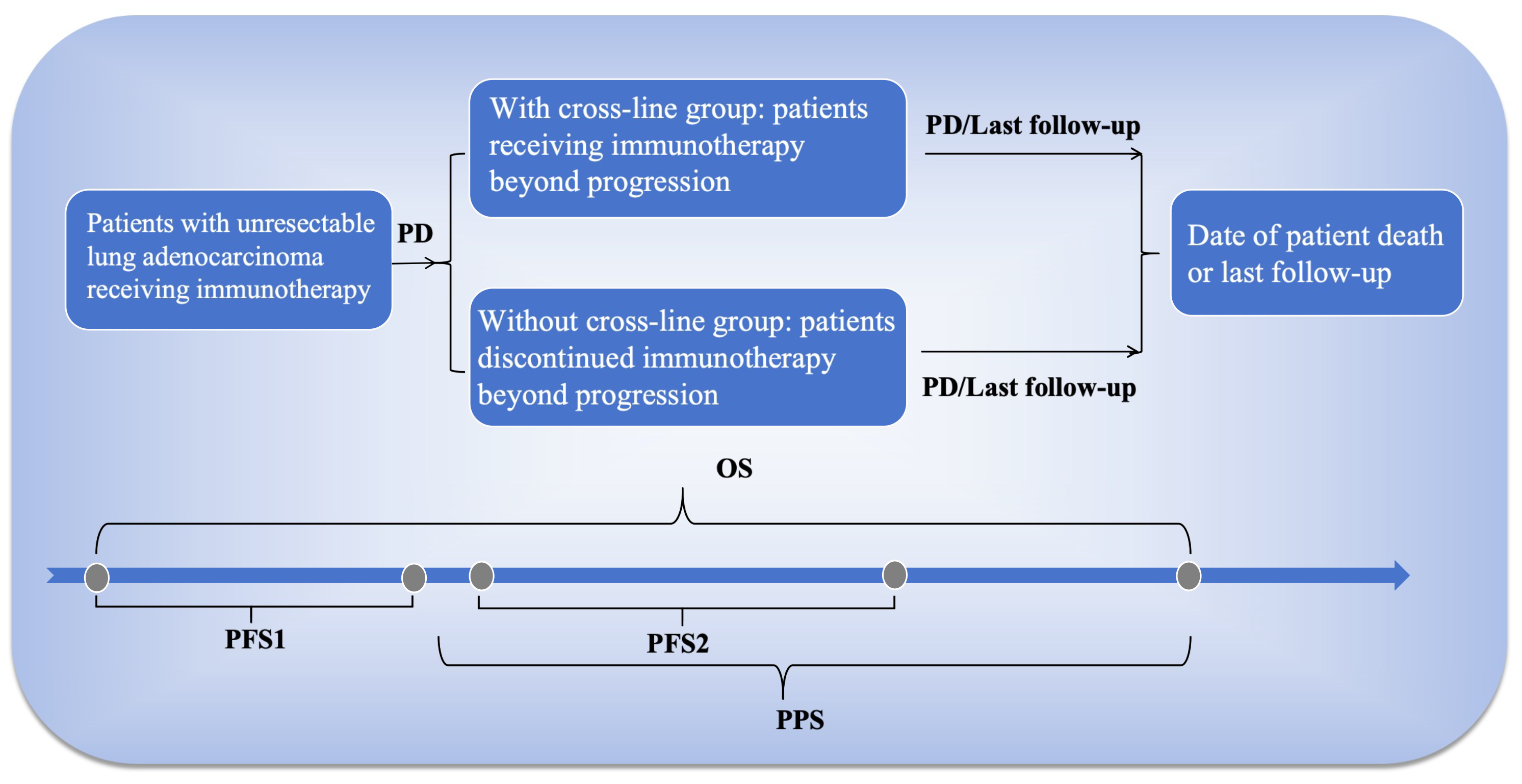

Supplement: Supplementary Additional Figure 1 — Study design illustrating the grouping strategy and survival endpoints. PD, progressive disease; PFS, progression-free survival; PPS, post-progression survival; OS, overall survival. [file Image1.tif]

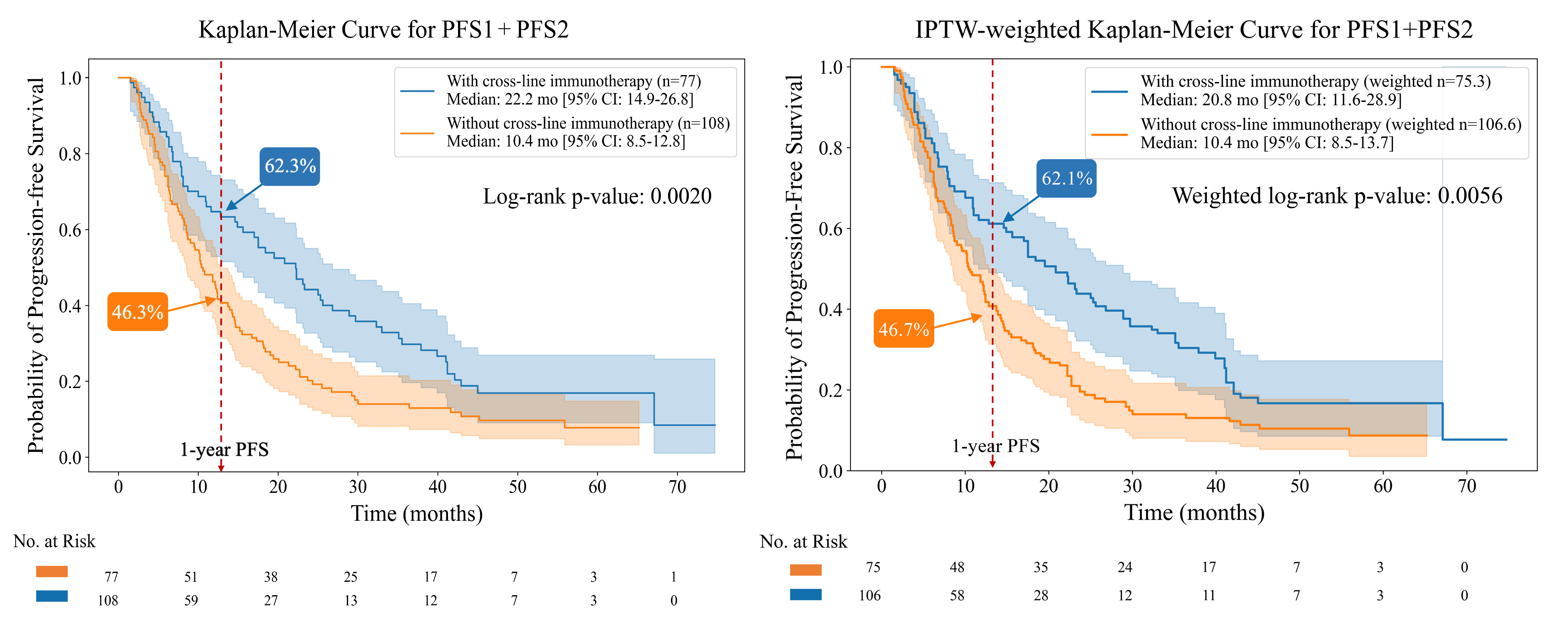

Supplement: Supplementary Additional Figure 2 — Kaplan–Meier survival analyses comparing cross-line immunotherapy (CIT) and without cross-line immunotherapy (non-CIT) groups for PFS1+ PFS2 before and after IPTW adjustment. IPTW, inverse probability of treatment weighting; PFS, progression-free survival; CI, confidence interval. [file Image2.tif]

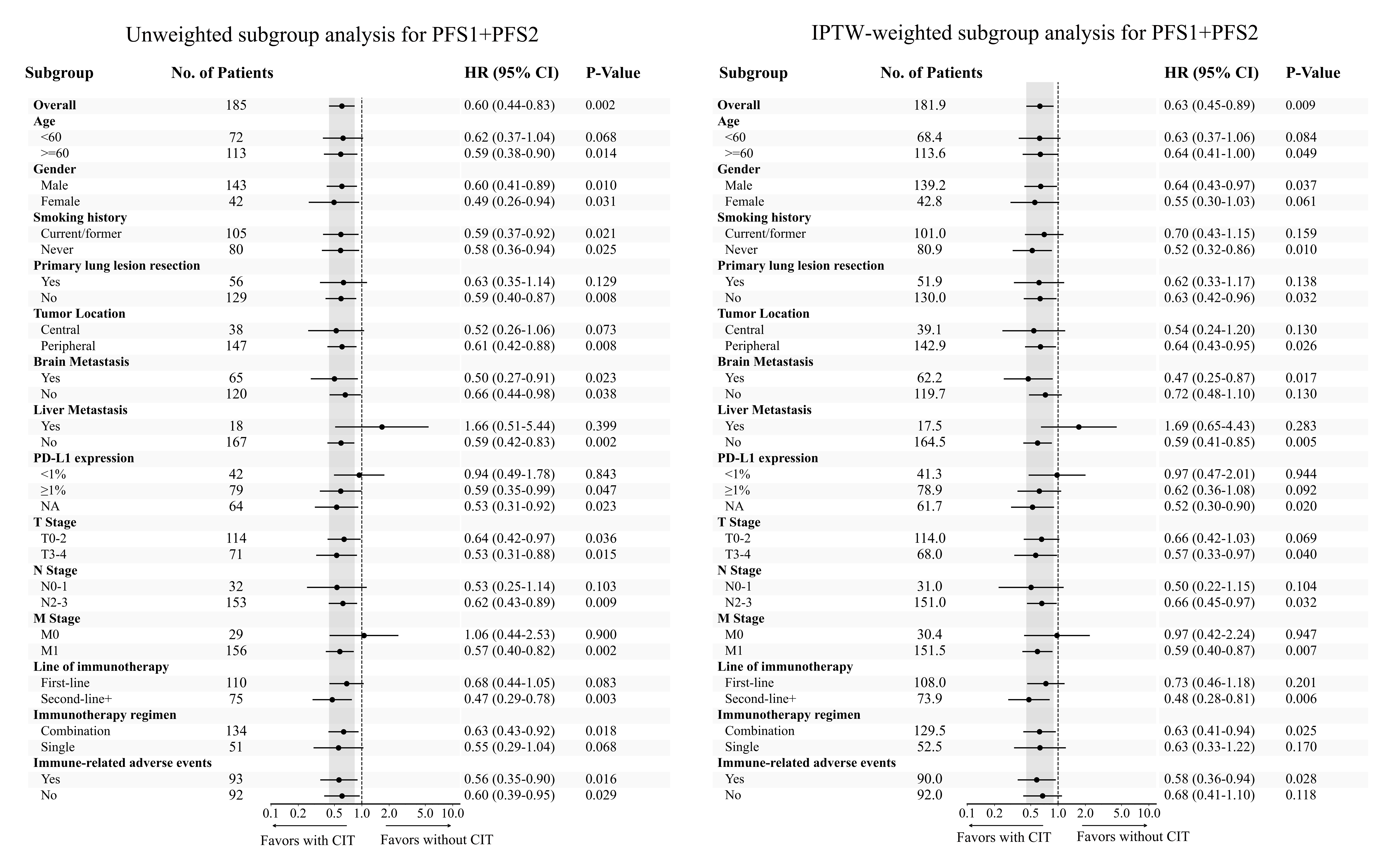

Supplement: Supplementary Additional Figure 3 — Kaplan–Meier survival analyses comparing cross-line immunotherapy (CIT) and without cross-line immunotherapy (non-CIT) groups for PFS1+ PFS2 before and after IPTW adjustment. IPTW, inverse probability of treatment weighting; PFS, progression-free survival; CI, confidence interval. [file Image3.tif]

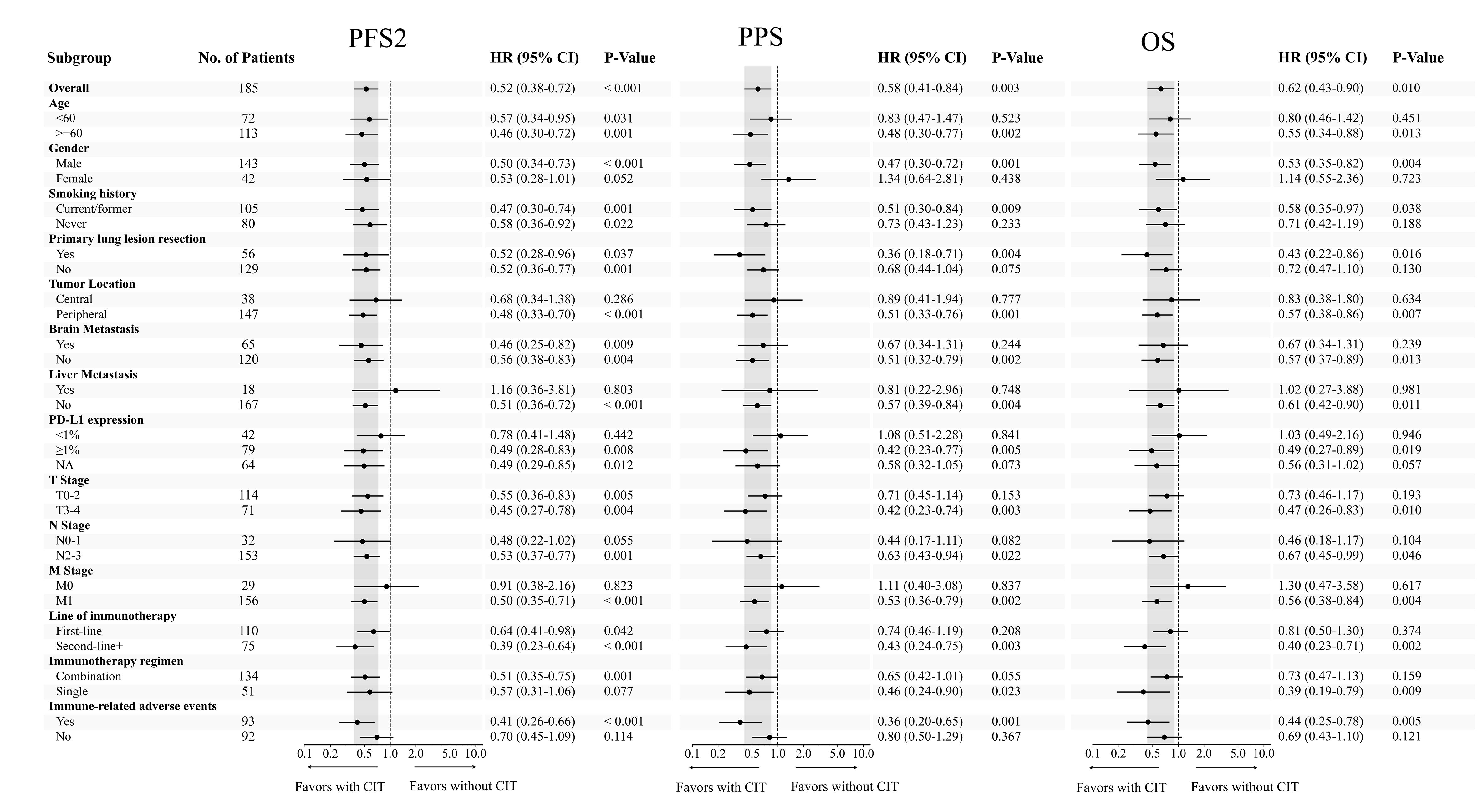

Supplement: Supplementary Additional Figure 4 — Kaplan–Meier survival analyses comparing cross-line immunotherapy (CIT) and without cross-line immunotherapy (non-CIT) groups for PFS1+ PFS2 before and after IPTW adjustment. IPTW, inverse probability of treatment weighting; PFS, progression-free survival; CI, confidence interval. [file Image4.tif]
