# Supplementary material for: Prognostic significance of continuing immunotherapy beyond progression in unresectable lung adenocarcinoma
Source: Front Immunol. 2026 Jul 8;17:1805604. doi: 10.3389/fimmu.2026.1805604 (PMC13388323; doi:10.3389/fimmu.2026.1805604)
Supplement: Supplementary file 5 [file Table1.docx]

Additional table 1. Overview of immunotherapeutic agents, dosages, and treatment schedules applied in this study.

| Drugs name | Target | Dose and usage | Administration method |
| --- | --- | --- | --- |
| Pembrolizumab | PD-1 | 200 mg, Q3W | Intravenous |
| Nivolumab | PD-1 | 240mg, Q2W | Intravenous |
| Tislelizumab | PD-1 | 200 mg, Q3W | Intravenous |
| Sintilimab | PD-1 | 200 mg, Q3W | Intravenous |
| Camrelizumab | PD-1 | 200 mg, Q3W | Intravenous |
| Atezolizumab | PD-L1 | 1200 mg, Q3W | Intravenous |
| Durvalumab | PD-L1 | 10 mg/kg, Q2W or 1500mg, Q4W | Intravenous |

Abbreviations: PD-1, programmed death-1; PD-L1, programmed death-ligand 1.

Additional table 2. Univariate Analyses of Clinical Parameters on PFS1+PFS2 Outcomes.

| **Variable** | **Before Weighting (PFS1+PFS2)** | | | **After Weighting (PFS1+PFS2)** | | |
| --- | --- | --- | --- | --- | --- | --- |
|  | Median Survival (95% CI) | HR (95% CI) | *P* | Median Survival (95% CI) | HR (95% CI) | *P* |
| **Cross-line immunotherapy** |  |  |  |  |  |  |
| CIT | 22.2 (14.9-26.8) | 0.60 (0.44-0.83) | 0.002 | 20.8 (11.6-28.9) | 0.63 (0.45-0.89) | 0.009 |
| Non-CIT | 10.4 (8.5-12.8) | 1.0 (Reference) | - | 10.4 (8.5-13.7) | 1.0 (Reference) | - |
| **Age** |  |  |  |  |  |  |
| ＜60 | 12.2 (8.7-15.2) | 1.02 (0.74-1.40) | 0.918 | 11.6 (8.3-14.6) | 1.00 (0.98-1.01) | 0.595 |
| ≥60 | 14.6 (10.6-18.4) | 1.0 (Reference) | - | 14.7 (10.9-18.4) | 1.0 (Reference) | - |
| **Gender** |  |  |  |  |  |  |
| Male | 14.2 (10.8-17.5) | 0.66 (0.46-0.95) | 0.026 | 12.8 (10.6-17.5) | 0.63 (0.47-0.86) | 0.004 |
| Female | 11.6 (7.9-19.5) | 1.0 (Reference) | - | 11.8 (7.9-19.5) | 1.0 (Reference) | - |
| **Smoking history** |  |  |  |  |  |  |
| Current/former | 15.6 (12.1-20.1) | 0.68 (0.49-0.93) | 0.015 | 14.6 (10.9-19.4) | 0.71 (0.51-0.99) | 0.045 |
| Never | 10.2 (7.8-13.9) | 1.0 (Reference) | - | 10.2 (8.2-15.2) | 1.0 (Reference) | - |
| **Primary lung lesion resection** |  |  |  |  |  |  |
| Yes | 11.6 (8.1-19.0) | 0.96 (0.68-1.35) | 0.795 | 11.6 (7.9-18.1) | 0.98 (0.67-1.44) | 0.933 |
| No | 14.2 (10.8-17.3) | 1.0 (Reference) | - | 13.9 (10.8-17.5) | 1.0 (Reference) | - |
| **Tumor location** |  |  |  |  |  |  |
| Peripheral | 12.8 (10.3-16.7) | 0.89 (0.61-1.30) | 0.544 | 12.3 (10.2-15.5) | 0.90 (0.64-1.27) | 0.563 |
| Central | 14.3 (8.3-22.7) | 1.0 (Reference) | - | 15.6 (8.3-22.2) | 1.0 (Reference) | - |
| **Brain Metastasis** |  |  |  |  |  |  |
| Yes | 12.8 (9.0-17.3) | 1.08 (0.78-1.49) | 0.663 | 14.5 (10.4-18.2) | 0.95 (0.67-1.33) | 0.762 |
| No | 13.7 (10.0-18.4) | 1.0 (Reference) | - | 12.4 (8.7-17.5) | 1.0 (Reference) | - |
| **Liver Metastasis** |  |  |  |  |  |  |
| Yes | 10.1 (4.6-17.5) | 1.70 (1.02-2.82) | 0.041 | 8.0 (3.9-17.5) | 1.80 (1.11-2.92) | 0.017 |
| No | 14.3 (10.9-17.5) | 1.0 (Reference) | - | 13.7 (10.8-17.5) | 1.0 (Reference) | - |
| **PD-L1 expression** |  |  |  |  |  |  |
| TPS＜1% | 8.3 (6.4-13.7) | 1.0 (Reference) | - | 8.3 (6.3-13.7) | 1.0 (Reference) | - |
| TPS≥1% | 14.6 (10.8-19.4) | 0.49 (0.33-0.72) | <0.001 | 14.3 (10.9-23.1) | 0.48 (0.33-0.70) | <0.001 |
| Unknown | 15.5 (8.1-22.3) | 0.52 (0.35-0.79) | 0.002 | 14.7 (8.1-22.2) | 0.56 (0.38-0.83) | 0.004 |
| **T Stage** |  |  |  |  |  |  |
| T0-2 | 14.6 (11.8-19.4) | 0.79 (0.57-1.09) | 0.145 | 14.6 (11.0-21.3) | 0.78 (0.55-1.09) | 0.141 |
| T3-4 | 10.8 (7.8-15.5) | 1.0 (Reference) | - | 10.8 (7.5-14.7) | 1.0 (Reference) | - |
| **N stage** |  |  |  |  |  |  |
| N0-1 | 13.7 (8.1-22.3) | 0.97 (0.64-1.46) | 0.872 | 13.7 (7.7-22.3) | 0.96 (0.61-1.52) | 0.855 |
| N2-3 | 12.8 (10.4-17.5) | 1.0 (Reference) | - | 12.4 (10.4-17.5) | 1.0 (Reference) | - |
| **M stage** |  |  |  |  |  |  |
| M0 | 22.2 (12.4-32.3) | 0.58 (0.37-0.92) | 0.021 | 22.2 (12.4-32.3) | 0.58 (0.38-0.88) | 0.010 |
| M1 | 11.8 (9.5-14.7) | 1.0 (Reference) | - | 10.9 (8.6-14.5) | 1.0 (Reference) | - |
| **Line of immunotherapy** |  |  |  |  |  |  |
| First-line | 14.6 (11.0-19.0) | 0.84 (0.62-1.16) | 0.295 | 14.6 (11.0-19.4) | 0.84 (0.60-1.17) | 0.293 |
| Second-line or later | 10.9 (7.9-15.6) | 1.0 (Reference) | - | 10.9 (7.9-15.6) | 1.0 (Reference) | - |
| **Immunotherapy regimen** |  |  |  |  |  |  |
| Combination therapy | 15.5 (12.2-20.1) | 0.67 (0.48-0.95) | 0.025 | 15.5 (12.1-20.1) | 0.68 (0.46-1.00) | 0.047 |
| Monotherapy | 8.1 (5.8-12.4) | 1.0 (Reference) | - | 8.1 (5.8-11.8) | 1.0 (Reference) | - |
| **irAEs** |  |  |  |  |  |  |
| Yes | 18.4 (14.9-23.3) | 0.58 (0.42-0.79) | 0.001 | 18.2 (14.6-23.3) | 0.60 (0.42-0.84) | 0.003 |
| No | 8.2 (7.4-10.6) | 1.0 (Reference) | - | 8.3 (6.8-11.0) | 1.0 (Reference) | - |

Additional table 3. Univariate Analyses of Clinical Parameters on PPS and OS Outcomes before weighting.

|  | **PPS** | | | **OS** | | |
| --- | --- | --- | --- | --- | --- | --- |
| **Variable** | Median Survival (95% CI) | HR (95% CI) | *P* | Median Survival (95% CI) | HR (95% CI) | *P* |
| **Cross-line immunotherapy** |  |  |  |  |  |  |
| CIT | 22.3 (15.0-30.1) | 0.58 (0.41-0.84) | 0.003 | 38.0 (26.5-51.1) | 0.62 (0.43-0.90) | 0.010 |
| Non-CIT | 9.1 (5.2-13.1) | 1.0 (Reference) | - | 18.9 (13.7-23.1) | 1.0 (Reference) | - |
| **Age** |  |  |  |  |  |  |
| ＜60 | 16.3 (11.7-35.7) | 0.75 (0.53-1.08) | 0.127 | 35.2 (20.0-47.0) | 0.84 (0.58-1.20) | 0.334 |
| ≥60 | 12.8 (8.8-18.3) | 1.0 (Reference) | - | 22.7 (17.1-26.9) | 1.0 (Reference) | - |
| **Gender** |  |  |  |  |  |  |
| Male | 12.4 (9.1-15.1) | 1.21 (0.80-1.82) | 0.374 | 20.4 (16.2-28.1) | 1.03 (0.68-1.56) | 0.885 |
| Female | 20.9 (14.5-28.8) | 1.0 (Reference) | - | 29.7 (23.1-41.2) | 1.0 (Reference) | - |
| **Smoking history** |  |  |  |  |  |  |
| Current/former | 11.3 (9.1-15.1) | 1.04 (0.73-1.48) | 0.828 | 21.0 (15.9-45.6) | 0.90 (0.63-1.28) | 0.561 |
| Never | 18.3 (13.8-25.3) | 1.0 (Reference) | - | 26.8 (20.1-34.7) | 1.0 (Reference) | - |
| **Primary lung lesion resection** |  |  |  |  |  |  |
| Yes | 19.9 (10.5-26.2) | 0.95 (0.65-1.39) | 0.789 | 28.1 (20.1-45.6) | 0.97 (0.66-1.43) | 0.892 |
| No | 13.3 (9.9-15.6) |  | - | 22.4 (16.2-29.7) | 1.0 (Reference) | - |
| **Tumor location** |  |  |  |  |  |  |
| Peripheral | 15.0 (10.8-20.9) | 1.02 (0.66-1.57) | 0.926 | 25.7 (19.0-35.2) | 1.00 (0.65-1.54) | 0.994 |
| Central | 11.3 (5.7-43.7) | 1.0 (Reference) | - | 23.1 (14.3-45.9) | 1.0 (Reference) | - |
| **Brain Metastasis** |  |  |  |  |  |  |
| Yes | 10.8 (6.2-15.6) | 1.01 (0.70-1.47) | 0.953 | 18.8 (13.5-45.6) | 1.03 (0.71-1.49) | 0.886 |
| No | 15.4 (12.4-21.8) | 1.0 (Reference) | - | 26.8 (21.0-38.0) | 1.0 (Reference) | - |
| **Liver Metastasis** |  |  |  |  |  |  |
| Yes | 11.0 (2.5-25.8) | 1.37 (0.77-2.43) | 0.286 | 15.0 (7.4-42.0) | 1.47 (0.82-2.61) | 0.192 |
| No | 14.5 (10.8-20.7) | 1.0 (Reference) | - | 25.7 (20.1-34.7) | 1.0 (Reference) | - |
| **PD-L1 expression** |  |  |  |  |  |  |
| TPS＜1% | 8.8 (4.3-29.2) | 1.0 (Reference) | - | 13.2 (9.4-40.0) | 1.0 (Reference) | - |
| TPS≥1% | 13.8 (10.5-18.3) | 0.86 (0.55-1.36) | 0.526 | 26.8 (18.2-42.0) | 0.72 (0.45-1.13) | 0.150 |
| Unknown | 18.4 (11.4-26.2) | 0.82 (0.51-1.32) | 0.416 | 26.9 (20.1-45.9) | 0.73 (0.46-1.17) | 0.193 |
| **T Stage** |  |  |  |  |  |  |
| T0-2 | 15.1 (11.3-25.3) | 0.82 (0.57-1.17) | 0.276 | 29.4 (21.0-45.6) | 0.76 (0.53-1.09) | 0.136 |
| T3-4 | 12.8 (8.1-16.3) | 1.0 (Reference) | - | 20.1 (15.0-26.5) | 1.0 (Reference) | - |
| **N stage** |  |  |  |  |  |  |
| N0-1 | 21.8 (9.9-47.2) | 0.68 (0.42-1.11) | 0.122 | 42.0 (18.8-57.7) | 0.76 (0.47-1.24) | 0.272 |
| N2-3 | 13.1 (10.1-17.5) | 1.0 (Reference) | - | 23.1 (18.2-29.7) | 1.0 (Reference) | - |
| **M stage** |  |  |  |  |  |  |
| M0 | 20.9 (10.8-inf) | 0.60 (0.35-1.01) | 0.055 | 38.0 (22.7-inf) | 0.56 (0.33-0.95) | 0.033 |
| M1 | 13.1 (9.9-16.3) | 1.0 (Reference) | - | 22.4 (18.2-27.7) | 1.0 (Reference) | - |
| **Line of Immunotherapy** |  |  |  |  |  |  |
| First-line | 13.8 (10.1-18.3) | 1.02 (0.71-1.45) | 0.920 | 25.7 (18.9-40.0) | 0.90 (0.63-1.29) | 0.573 |
| Second-line or later | 15.6 (9.7-25.8) | 1.0 (Reference) | - | 24.3 (15.9-35.2) | 1.0 (Reference) | - |
| **Immunotherapy regimen** |  |  |  |  |  |  |
| Combination therapy | 15.0 (12.4-21.8) | 0.77 (0.53-1.12) | 0.174 | 27.7 (21.0-41.2) | 0.68 (0.47-0.98) | 0.041 |
| Monotherapy | 11.0 (5.2-20.7) | 1.0 (Reference) | - | 18.0 (10.9-26.8) | 1.0 (Reference) | - |
| **irAEs** |  |  |  |  |  |  |
| Yes | 22.3 (13.1-35.7) | 0.58 (0.41-0.83) | 0.003 | 45.6 (27.9-51.1) | 0.53 (0.37-0.75) | <0.001 |
| No | 10.5 (5.4-14.4) | 1.0 (Reference) | - | 15.9 (11.7-21.0) | 1.0 (Reference) | - |

Abbreviations: PPS, post-progression survival; OS, overall survival; CI, confidence interval; HR, Hazard Ratio; CIT, with cross-line immunotherapy; Non-CIT, without cross-line immunotherapy; PD-L1, programmed cell death-ligand 1; TPS, tumor proportion score; T stage, tumor stage; N stage, lymph node stage; M stage, distant metastasis stage; irAEs, immune-related adverse events.

Additional table 4. Multivariate Analyses of Clinical Parameters on PFS and OS Outcomes before weighting.

|  | **PFS2** | | **PFS1+PFS2** | | **PPS** | | **OS** | |
| --- | --- | --- | --- | --- | --- | --- | --- | --- |
| **Variable** | HR (95%CI) | *P* | HR (95%CI) | *P* | HR (95%CI) | *P* | HR (95%CI) | *P* |
| **Cross-line immunotherapy** |  |  |  |  |  |  |  |  |
| CIT vs. Non-CIT | 0.58 (0.41-0.81) | 0.001 | 0.59 (0.41-0.83) | 0.003 | 0.65 (0.45-0.94) | 0.022 | 0.65 (0.45-0.94) | 0.021 |
| **Gender** |  |  |  |  |  |  |  |  |
| Male vs. Female | 0.86 (0.53-1.41) | 0.552 | 1.00 (0.61-1.64) | 0.985 | 1.64 (0.95-2.84) | 0.076 | 1.65 (0.95-2.85) | 0.076 |
| **Smoking history** |  |  |  |  |  |  |  |  |
| Current/former vs. Never | 0.94 (0.62-1.43) | 0.789 | 0.73 (0.48-1.10) | 0.132 | 0.94 (0.59-1.48) | 0.782 | 0.81 (0.52-1.27) | 0.364 |
| **Liver Metastasis** |  |  |  |  |  |  |  |  |
| Yes vs. No | 1.02 (0.59-1.78) | 0.933 | 1.25 (0.73-2.16) | 0.419 | 1.40 (0.75-2.61) | 0.291 | 1.43 (0.77-2.65) | 0.264 |
| **PD-L1 expression** |  |  |  |  |  |  |  |  |
| TPS≥1% vs. TPS＜1% | 0.60 (0.40-0.92) | 0.018 | 0.52 (0.34-0.78) | 0.002 | 0.89 (0.56-1.44) | 0.641 | 0.73 (0.45-1.16) | 0.183 |
| Unknown vs. TPS＜1% | 0.53 (0.34-0.84) | 0.006 | 0.47 (0.30-0.72) | 0.001 | 0.69 (0.42-1.23) | 0.142 | 0.58 (0.36-0.96) | 0.033 |
| **M stage** |  |  |  |  |  |  |  |  |
| M0 vs. M1 | 0.52 (0.32-0.84) | 0.008 | 0.57 (0.35-0.91) | 0.019 | 0.56 (0.33-0.96) | 0.040 | 0.53 (0.31-0.92) | 0.023 |
| **Immunotherapy regimen** |  |  |  |  |  |  |  |  |
| Combination therapy vs. Monotherapy | 0.62 (0.43-0.89) | 0.010 | 0.51 (0.35-0.73) | <0.001 | 0.79 (0.54–1.17) | 0.243 | 0.65 (0.44-0.95) | 0.026 |
| **irAEs** |  |  |  |  |  |  |  |  |
| Yes vs. No | 0.64 (0.46-0.89) | 0.009 | 0.52 (0.37-0.73) | <0.001 | 0.51 (0.35-0.74) | 0.001 | 0.44 (0.30-0.64) | <0.001 |

Abbreviations: PFS, progression-free survival; PPS, post-progression survival; OS, overall survival; CI, confidence interval; HR, Hazard Ratio; CIT, with cross-line immunotherapy; Non-CIT, without cross-line immunotherapy; PD-L1, programmed cell death-ligand 1; TPS, tumor proportion score; M stage, distant metastasis stage; irAEs, immune-related adverse events.

Additional table 5. Variance Inflation Factor Analysis of Nomogram Variables after IPTW.

| **Variable** | **VIF** | **Interpretation** |
| --- | --- | --- |
| Sex | 1.10 | No multicollinearity |
| Liver metastasis | 1.10 | No multicollinearity |
| PD-L1 expression status | 1.26 | No multicollinearity |
| Cross-line immunotherapy | 1.00 | No multicollinearity |
| M stage | 1.05 | No multicollinearity |
| Treatment regimen | 1.05 | No multicollinearity |
| Immune-related adverse events | 1.04 | No multicollinearity |

Abbreviations: IPTW, inverse probability of treatment weighting; VIF, variance inflation factor. VIF values > 2.0 indicate potential multicollinearity concerns; PD-L1, programmed death-ligand 1.
